# Poly (A)^+^ Transcriptome Assessment of *ERBB2*-Induced Alterations in Breast Cell Lines

**DOI:** 10.1371/journal.pone.0021022

**Published:** 2011-06-22

**Authors:** Dirce Maria Carraro, Elisa Napolitano Ferreira, Gustavo de Campos Molina, Renato David Puga, Eduardo Fernandes Abrantes, Adriana Priscila Trapé, Bedrich L. Ekhardt, Diana Noronha Nunes, Maria Mitzi Brentani, Wadih Arap, Renata Pasqualini, Helena Brentani, Emmanuel Dias-Neto, Ricardo Renzo Brentani

**Affiliations:** 1 Centro Internacional de Ensino e Pesquisa, Hospital AC Camargo, São Paulo, São Paulo, Brazil; 2 Instituto de Biociências, Universidade de São Paulo, São Paulo, São Paulo, Brazil; 3 Faculdade de Medicina, Universidade de São Paulo, São Paulo, São Paulo, Brazil; 4 David H. Koch Center, The University of Texas M. D. Anderson Cancer Center, Houston, Texas, United States of America; Instituto Butantan, Brazil

## Abstract

We report the first quantitative and qualitative analysis of the poly (A)^+^ transcriptome of two human mammary cell lines, differentially expressing (human epidermal growth factor receptor) an oncogene over-expressed in approximately 25% of human breast tumors. Full-length cDNA populations from the two cell lines were digested enzymatically, individually tagged according to a customized method for library construction, and simultaneously sequenced by the use of the Titanium 454-Roche-platform. Comprehensive bioinformatics analysis followed by experimental validation confirmed novel genes, splicing variants, single nucleotide polymorphisms, and gene fusions indicated by RNA-seq data from both samples. Moreover, comparative analysis showed enrichment in alternative events, especially in the exon usage category, in *ERBB2* over-expressing cells, data indicating regulation of alternative splicing mediated by the oncogene. Alterations in expression levels of genes, such as *LOX*, *ATP5L*, *GALNT3*, and *MME* revealed by large-scale sequencing were confirmed between cell lines as well as in tumor specimens with different *ERBB2* backgrounds. This approach was shown to be suitable for structural, quantitative, and qualitative assessment of complex transcriptomes and revealed new events mediated by *ERBB2* overexpression, in addition to potential molecular targets for breast cancer that are driven by this oncogene.

## Introduction

Global comparative analysis of transcriptomes is the most effective approach for definition of alterations in gene expression profiles and has led to the identification of key defective elements involved in complex diseases such as cancer. Different aspects of quantitative gene expression have been investigated in breast cancer by microarray-based analysis [1]–[6], with important implications for prognosis [7], [8]. At present, the management of breast cancer patients takes into consideration a combination of clinical and histopathological characteristics, together with the measurement of estrogen (ER) and progesterone (PR) hormone receptors and Her2/*ERBB2* overexpression/amplification.

ERBB2 (human epidermal growth factor receptor) is a member of the tyrosine kinase receptor family, and its amplification has long been considered to play a crucial role in the tumorigenic process [9], [10]. *ERBB2*, overexpressed in 25 to 30% of human breast cancers [11], [12], is associated with metastasis [13], and *ERBB2*-overexpressing cells are self-sufficient with respect to, anchorage-independent growth and efficient in invasion [14].

Patients bearing *ERBB2*-overexpressing tumors are usually treated with Trastuzumab (Herceptin®), a therapeutic monoclonal antibody against ERBB2. However, a significant fraction (∼60%) of patients with metastatic breast tumors does not respond to the treatment [15], highlighting the necessity for continued investigation of *ERBB2*-mediated modifications in breast cells.

In light of the importance of *ERBB2* in breast cancer, HB4a [16] and HB4aC5.2 [17], the parental and the *ERBB2* overexpressing cell lines, respectively, have been used to investigate quantitative transcriptional alterations in mammary cells mediated by *ERBB2* overexpression [18], [19]. However, given the high complexity of the mammalian transcriptome [20], [21] the use of more sensitive approaches that enable assessment of not only quantitative but also qualitative aspects of the transcriptome has presently been intensified. In this sense, clear consensus has emerged that next-generation sequencing (NGS), which provides digital-counting of the transcriptome, is more advantageous than other solely quantitative methodologies [22]–[26].

Here we present the first NGS-based qualitative and quantitative evaluation of the mammary cell transcriptome modulated by *ERBB2* over-expression. By combining *Dpn*II-restriction and parallel-tagged sequencing, we performed analysis of the poly (A)^+^ transcriptomes of two human mammary cell lines: HB4a [17] and its *ERBB2*-overexpressing clone, C5.2 [16]. Whereas no qualitative aspects were correlated with ERBB2 over-expression, significant enrichment of alternative splicing events was shown to be mediated by the overexpression of this oncogene. Additionally, novel *ERBB2*-driven genes and transcript variants were revealed in these cell lines and were also validated in tumor specimens with high ERBB2 expression. Moreover, novel genes, splicing variants, single nucleotide polymorphisms (SNPs) and gene fusions were detected in sequences from both cell lines, data contributing further to the definition of the human transcriptome.

## Results

### Whole transcriptome assessment of HB4a and C5.2 cell lines

**Figure 1:**
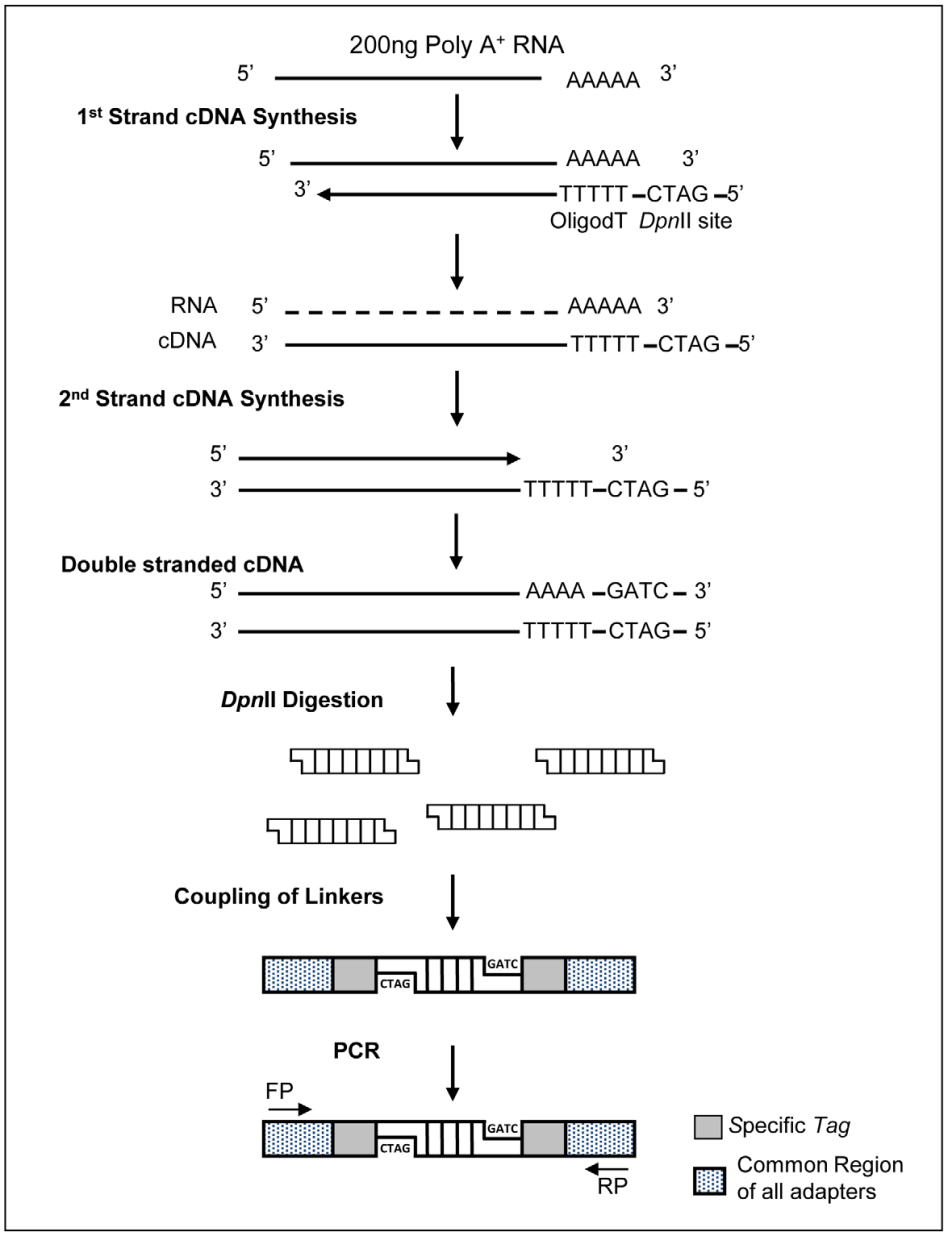
Schematic representation of cDNA libraries. An oligo-dT primer containing the *Dpn*II restriction site was used for first-strand synthesis. Second-strand synthesis was performed with RNase H, DNA polymerase and T4 DNA ligase. The double-stranded cDNA was digested with *Dpn*II, followed by coupling of linkers and PCR.

**Figure 2:**
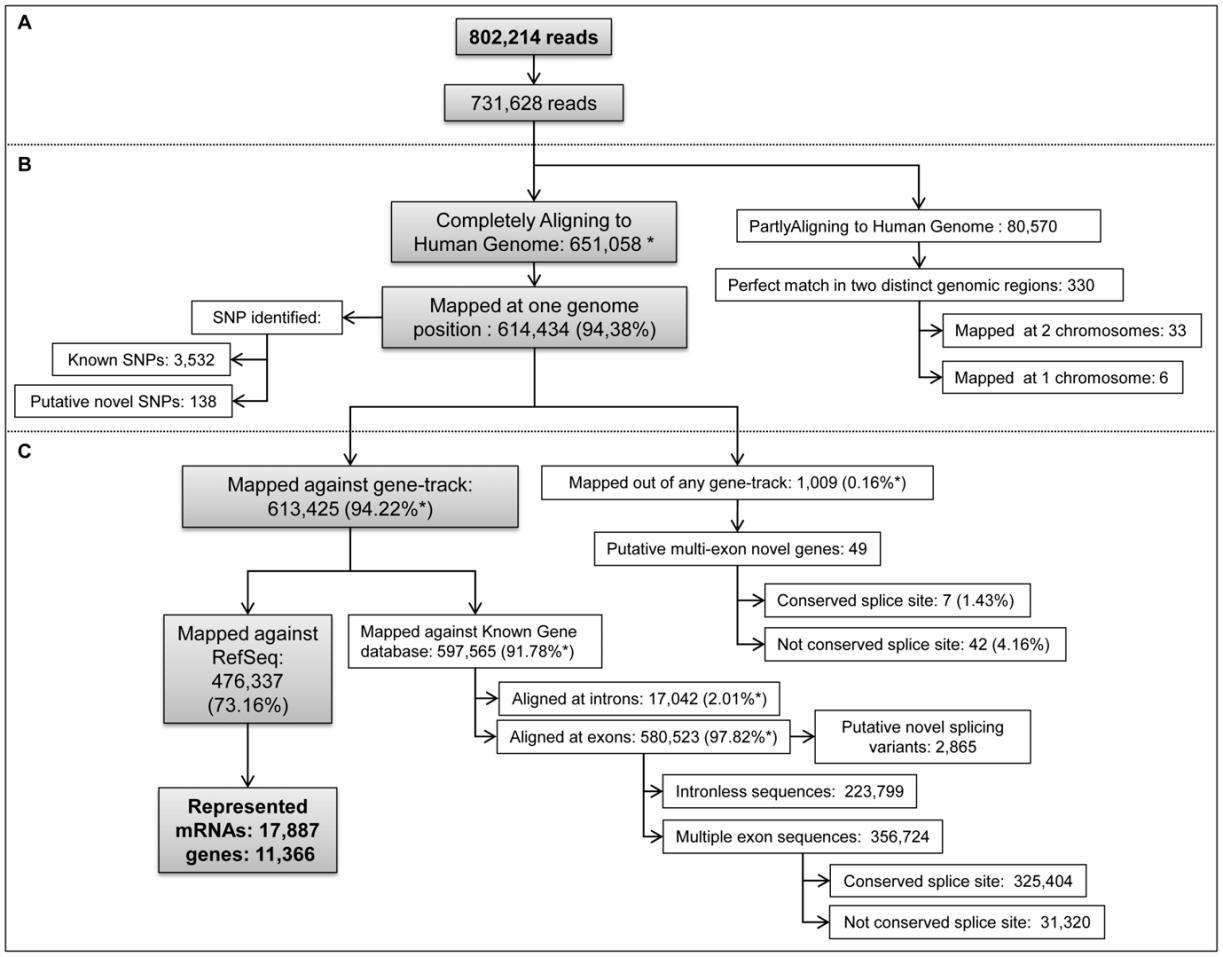
Bioinformatics analyzes flowchart. (A) Initial filters. (B) Human genome alignment. Completely Aligning to Human Genome corresponds to reads that aligned to the genome sequence using the criteria of coverage ≥70% and identity ≥96%. Sequences aligning to more than one genome region following these criteria were discarded. Single-hit high coverage genome alignment sequences were used for discovery of novel SNPs. Partially Aligning to Human Genome corresponds to reads that aligned to genome sequence using the criteria of coverage ≥20% and ≤80% and identity ≥99.9%. These reads were used for discovery of gene fusion events. (C) Transcript databases alignment. For transcriptome analysis, reads from completely aligning sub-set were further aligned to known gene databases for the discovery of novel splicing variants. Additionally, reads were aligned to RefSeq databases for obtaining the number of transcripts (mRNAs) and genes identified.

A customized method for simultaneous sequencing of the poly (A)^+^ transcriptome of multiple samples was established. Double-stranded cDNA converted from purified poly A^+^ mRNA from HB4a and C5.2 cell lines was prepared and digested with the frequent cutter Dpn*II*. Adapters containing specific 4nt- barcode were designed and added to *Dpn*II digested cDNAs from both cell lines, and samples were pooled before sequencing (Figure 1). cDNA samples were sequenced on the Titanium 454-Roche platform generating 802,214 reads, with a total of 160,223,981 high-quality nucleotides (Phred ≥20) (Figure 2). The mean size of reads was 199.7±66.72. Sequence reads were submitted to The Sequence Read Archive (SRA) (accession number SRA012436.2).

Sequences were filtered to exclude mitochondrial DNA and ribosomal RNAs, as well as sequences without adapters, yielding 731,628 reads (91.2%) (Figure 2A). From this, two subsets were separated according to genome alignment parameters that differed in percentage of coverage and identity: a subset of completely aligning reads and another subset of reads with partial alignment to the human genome, respectively containing 651,058 reads (89%) (coverage ≥70% and identity ≥96%) and 80,570 reads (11%) (coverage ≥20% and ≤80% and identity ≥99.9%). From the former, 614,434 (94.4%) that present single-hit matches (Figure 2B) in the genome were used for gene coverage and identification of novel genes, splicing variants and SNPs. From the latter subset, reads that present perfect matches in two genomic regions were used to assess gene-fusion events.

### Gene coverage and novel genes

**Figure 3:**
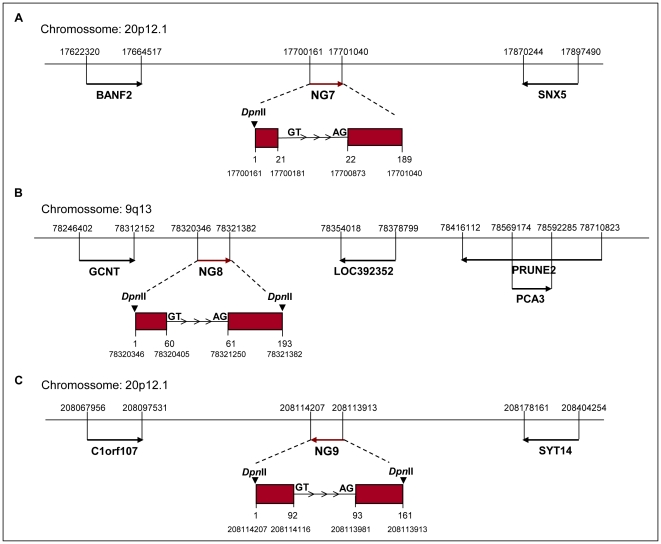
Genomic coordinates of the partial sequences of novel human genes. Arrows represent the genomic localization of each gene and its transcription orientation. The red arrows represent the novel genes [(A) NG7; (B) NG8; (C) NG9]. In an expanded view, the genomic coordinates of the NGs are shown, as well as the conserved splice sites depicted in the introns and the *Dpn*II restriction sites. Genomic representations are not scaled.

A small fraction of 1,009 (0.16%) reads mapping out of any gene-track position was explored to find novel human genes (Figure 2C). Most of these sequences (960 reads) were single continuous hits, and only a minority (49 sequences) was derived from multiple-exon hits. Six out of the 49 multi-exon genes exhibited canonical splice sites at their putative introns and were selected for validation by the use of cDNA from these cell lines, and three (50%) new human transcripts were thereby confirmed. The minimal distance between the novel transcribed regions and the known genes in the vicinity was 8 Kb (Figure 3), suggesting that the three confirmed transcripts are not extensions of the known genes.

From the 613,425 reads (98.83%) that could be mapped against at least one gene track (Figure 2B), 476,337 reads (73.16%) were aligned to RefSeq (Figure 2C) that demonstrated a expression of 38.9% (17,887 entries) of the complete RefSeq transcript repertoire (build 36) in these cell lines. Given that 17,887 full-length mRNAs correspond to 60,500,115 nt, the base-pair representation was 23.48% (14,208,089 nt covered).

To estimate transcript representation we verified the read distribution throughout the entire full-length mRNAs by calculation of their relative position as described [27]. A slightly higher concentration of sequences was seen in the central portion of the transcripts (Figure S1A). The 5′ end of full transcripts was well-represented, irrespective of the original transcript lengths (Figure S1B). This fact shows high-quality RNA templates, efficient cDNA synthesis and library construction methodology with no biased representation of short transcripts.

### Single nucleotide polymorphism

The 614,434 reads mapping at a single genome position were used for assessment of novel SNPs (Figure 2B). According to a set of parameters and criteria (see Methods), 3,532 known SNPs and 138 (3.8%) potentially novel SNPs were thereby revealed.

Eighteen putative SNPs were subjected to validation by Sanger-sequencing of genomic DNA from both cell lines. A high validation rate (89%) was obtained and revealed 16 new SNPs (12 heterozygous and 4 homozygous SNPs) (Table S1 and Figure S2). Nine of the 16 new SNPs were located in coding regions, and four resulted in non-synonymous amino acid substitutions, all predicted as benign substitutions by PolyPhen [28]. Intriguingly, the different chromatogram peak height of *DKK1* heterozygous SNP observed in C5.2 cells was suggestive of an apparent allelic expression imbalance in the *ERBB2*-overexpressing cell line.

### Novel splicing variants

**Figure 4:**
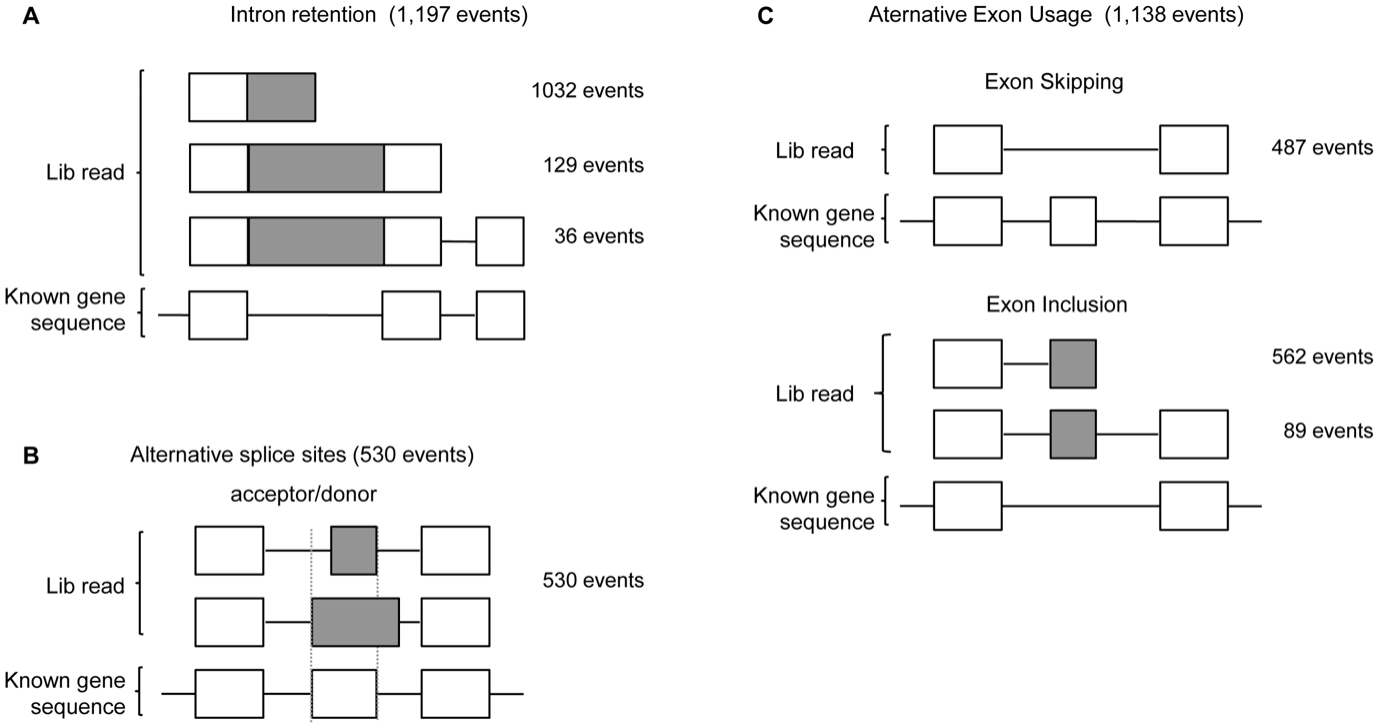
Putative new alternative splicing variants - The 2,865 novel alternative splicing events detected in our approach are distributed according to the type of event reported. White squares represent the known exons and grey squares represent the alternative exons. The number of events is shown on the right side of each event type. (A) Intron retention showing the presence of one or more constitutive exons. (B) Alternative splice donor or acceptor site usage. (C) Alternative exon usage events were sub-classified into exon skipping and exon inclusion events that show one or both flanking constitutive exons.

Putative novel splicing variants could be identified from the 597,565 reads that aligned against mRNA databases (Figure 2C). From them a total of 2,865 were potentially novel alternative splicing (AS) events and were distributed in distinct categories (Figure 4). For validation, we focused on the exon inclusion set (Figure 4C), because the feasibility of designing primers in the newly-included exons leads to more reliable results with regard to the expression of specific variants. From the subset of 89 events containing two known flanking exons (Figure 4C) 20 exon-inclusion events were selected for validation. From them, 18 (90%) new *bona fide* exon inclusion AS-variants (Figure S3) were confirmed.

### Gene fusion

From the partially aligning subset (Figure 2B), reads that presented perfect match in two distinct genomic regions were considered as potential gene fusion events. After removing 26 false-positive candidates detected by the presence of *Dpn*II restriction sites at the fusion junction (Figure S4), which indicates chimeric artifacts produced during library construction, 330 reads remained. Next, chimeras within the same gene and between a gene and its corresponding pseudogene were discarded. Finally, reads presenting partial alignment to unique exonic regions were selected resulting in a total of 39 gene fusion candidates, 33 inter and 6 intra-chromosomal events (Figure 2B). From the 39 gene fusion reads, 32 events presented micro-homology (27 inter- and 5 intra-chromosomal), and only one (inter-chromosomal) presented 2 bases that did not align to any of the regions involved in the fusion, named short non-templated sequences (Table S2).

**Figure 5:**
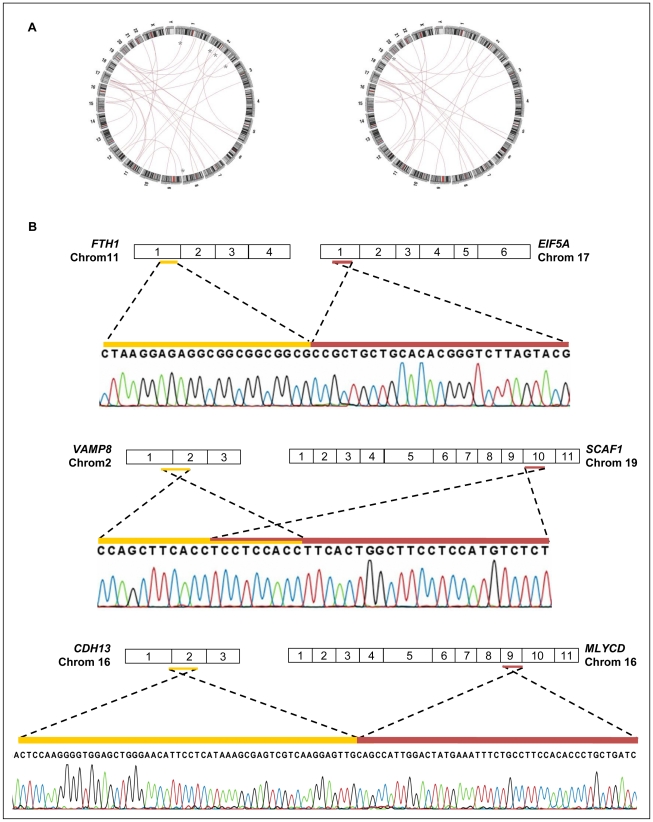
Identification and validation of gene fusion events. (A) Circus-plot representation of inter- and intra-chromossomal gene fusion identified for each cell line, C5.2 (left panel) and HB4a (right panel), (*) Inter-chromosomal gene fusion events reported exclusively by one of the cell lines. (B) Validation of 3 gene fusions. The exon distribution of the original genes is represented by the numbered squares, and the regions involved in the fusion are represented by the colored lines.

Ninety five percent of gene fusion events (37 out of 39) were reported by more than 2 reads and 72% (28 out of 39) were reported by both cell lines, suggesting that they are bona-fide gene fusion events (Figure 5A). However, only 3 out of 14 events randomly selected for validation (11 inter- and 3 intra-chromosomal) were confirmed by qRT-PCR assays using cDNA and genomic DNA from both cells (Figure 5B; Table S2). The two inter-chromosomal gene fusions (*FTH1*/chromosome 11 -*EIF5A*/chromosome 17; *VAMP8*/chromosome 2 - *SCAF1*/chromosome 19) were validated in both cDNA and genomic DNA from HB4a and C5.2 cells, a result that demonstrates their genomic rearrangement origin. Conversely, the intra-chromosomal gene fusion reported (*CDH13*/chromosome 16 – *MLYCD*/chromosome 16) was validated only in cDNA from C5.2 cells. The absence of amplification in genomic DNA suggests that this fusion might result from trans-splicing event or from genomic rearrangement, whose fragment size precludes its amplification by standard PCR conditions.

### *ERBB2*-mediated effects in the poly (A)^+^ transcriptome of a breast cell-line: assessment of qualitative and quantitative events

For comparison of both transcriptomes, a subset of 410,788 reads with more stringent criteria for tag assignment (see Methods) was used.

To check whether *ERBB2* overexpression could augment genome instability that could be reflected by gene fusion events in the transcriptome of both mammary cell lines, we compared the number of gene fusion events between *ERBB2*-basal (HB4a) and *ERBB2*-high expression (C5.2) cell lines normalized by the total number of reads of each respective cell. The normalized number obtained from each cell line was highly similar: 34.2 events in Hb4a cells and 38 events in C5.2 cells (p = 0.24). From the three validated gene fusions, *VAMP8/SCAF1* and *CDH13/MLYCD* were reported in RNAseq from C5.2 cells exclusively (Table S2). However *VAMP8/SCAF1* was detected in both cDNA and genomic DNA form HB4a and C5.2 cells, whereas *CDH13/MLYCD* confirmed to be exclusively expressed in cDNA of C5.2 cells. Based on our results, no conclusive evidence was found towards the influence of *ERBB2* overexpression on genomic instability.

To explore the influence of *ERBB2* overexpression on the regulation of alternative splicing, we compared the number of AS events of each category in each cell line (Table S3), normalized by the total number of reads obtained. An enrichment of alternative splicing events was observed in the C5.2 cells represented by the categories of exon skipping (p = 1.35E-6), exon inclusion (p = 0.005), and alternative acceptor/donor splice sites (p = 2.4E-7).

To confirm this enrichment, the expression of eight events out of 18 that were reported only by reads obtained with C5.2 specific tag, indicative of a specifically or highly expressed splicing variant in C5.2 cells, was evaluated by quantitative RT-PCR in both cell lines. Although none of these AS events was shown to be specific for C5.2 cells, since amplification was detected in both cell lines, six out of eight (75%) confirmed a higher expression in C5.2 cells (fold >2), a result indicating that the enrichment resulted from an increase in the expression level of the AS transcripts possibly influenced by *ERBB2* overexpression. Further evidence that this oncogene influenced specific AS variants of a gene is that for five out of six events, only the splicing variants evaluated seem to be influenced by *ERBB2* overexpression (Figure S3 and Table S4), i.e., a comparison between the number of entire gene-related sequences obtained from each cell line showed no difference in expression levels (or even higher expression in HB4a cells).

Five out of six splicing variants overexpressed in C5.2 are positioned within the coding sequence (CDS). For three variants the exon insertion resulted in premature stop codons (*KIAA1033*, *CSRP2BP* and *PRCC*) that might produce truncated proteins. The exon insertion in the variant of the *CLTC* gene generated an in-frame insertion of 7 amino acids, with no alteration in protein domains. For the *NR2C1* gene, the inserted exon resulted in a smaller protein isoform (177 amino acids are removed) that resulted in an alteration in the N-terminal portion with the loss of two domains, (the vitamin D and zinc-finger nuclear hormone receptor) and gain of the retinoid X receptor domain. If the putative non-functional protein plays a role in *ERBB2*-driven breast tumor remains to be addressed.

Gene expression analysis was carried out by counting the number of reads representing each gene, independently of their relative position within the full-length mRNA. Indeed, as expected, we have found more tags representing the *ERBB2* gene in C5.2 than in HB4 cells, with a 15-fold expression difference, a result indicating that RNA-Seq can provide informative and confident quantitative results.

**Figure 6:**
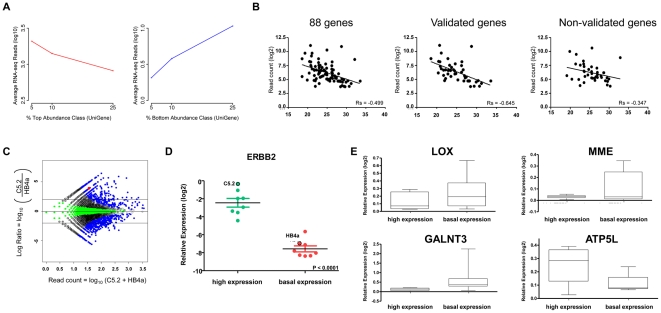
Gene expression analysis. (A) Correlation of Unigene cluster sizes classified by the ESTs abundance-classes: bottom 25%, 10% and 5% (left panel) and top 25%, 10% and 5% (right panel) by the average number of reads from the RNAseq (log10). (B) Correlation between number of reads of RNA-seq and Cycle threshold (Ct values) of qRT-PCR experiments for all, validated and non-validated gene set. (C) Relative gene expression between C5.2 and HB4a cells. The 2 black lines represent the cut-off value of log_2_ ratio ≥|2|- fold-change ≥|4|. The blue colored points correspond to genes with a Bayes Error Rate equal to 0.0. *ERBB2* relative expression is identified by the red point. (D) Classification of breast tumor samples in high and basal *ERBB2*-expression by relative quantification of *ERBB2* transcript. The black circled points represent the expression level of the cell lines with high (C5.2) and basal (HB4a) *ERBB2* expression levels. (E) Relative expression of the genes *LOX*, *MME*, *GALNT3* and *ATP5L* in the two groups of tumor breast samples with high and basal *ERBB2* expression.

To check if the transcript level is preserved by our RNA-seq we compared our RNAseq data to Unigene cluster-size, a measure of transcriptional abundance. We have selected Unigene clusters and grouped them according to the total number of ESTs for each class of abundance (top or bottom 25%, 10% and 5%). Then we calculated the average number of sequences obtained in RNAseq experiments for the respective set of genes, for each of these abundance-classes. RNAseq reads showed a positive correlation, where genes with larger Unigene-cluster sizes were sequenced much more often than genes with lower Unigene-cluster sizes (Figure 6A). Thus, we can conclude that transcriptional levels are indeed preserved by our RNAseq data.

A total of 436 potentially differentially expressed genes, 192 up-regulated and 244 down-regulated, was identified in C5.2 cells (Figure 6C). Eighty-eight of these were evaluated by qRT-PCR, and the differential expression of 46 genes (52.3%) was validated (Table S5).

To evaluate the correlation between gene expression quantification based on RNA-seq and qRT-PCR, a comparison between numbers of reads and Cycle threshold (CT) values was performed for 88 genes using TaqMan assays and p<0.01 was considered as statistically significant. This comparison showed a negative significant correlation when all genes (p<0.0001) or only the validated ones (p<0.0001) were analyzed (Figure 6B). Even for the non-validated genes a marginal significance was seen (p = 0,0263). This result reinforces the effectiveness of this methodology for parallel sequencing of two different and barcoded cDNA populations and the feasibility of our approach for the determination of gene expression profiles.

To investigate the biological properties of genes modulated by *ERBB2*-mediated expression, we classified the 46 genes with confirmed differential expression according to Biological Processes in Gene Ontology (GO) and also within KEGG database pathways. Using FunNet [29] we identified 17 enriched GO and 8 KEGG categories in both cell lines (Figure S5).

To determine whether these quantitative gene expression profiles could also be associated with human tumors over-expressing ERBB2, we investigated the expression levels of the 46 validated genes in 14 human breast ductal carcinoma samples containing high (7 samples) or basal levels (7 samples) of ERBB2 expression (Figure 6D). Despite tumor and patient heterogeneity, as well as the gap between cell line models and clinical samples, 4 genes (8.6%) were also modulated in breast tumor samples with distinct ERBB2 backgrounds: *ATP5L* was increased in the ERBB2-positive samples, whereas *LOX* (ENSG00000113083), *GALNT3* (ENSG00000115339), and *MME* (ENSG00000196549) showed reduced expression when ERBB2 was elevated (Figure 6E).

One of the most important signaling pathways driven by ERBB2 involves PI3K/AKT that activates the protein kinase mammalian target of rapamycin (mTOR), an important regulator of mRNA translation that controls cell proliferation [30]. To evaluate whether the genes modulated by *ERBB2*-mediated expression identified in this study were under mTOR transcriptional control, we treated both cell lines with rapamycin and investigated their expression after treatment. From the 46 validated genes, 19 (41.3%) showed reduction or inversion in relative fold-difference between C5.2/HB4a cells (Table S6). Some of these genes have already been reported to be modulated rapamycin treatment [31]–[34], strengthening the possibility that the additional genes, not previously reported, are also mTOR downstream regulated.

## Discussion

The approach presented here has provided data for quantitative and architectural aspects of two mammary cell-line poly (A)^+^ transcriptomes, and has shown the potential to fully represent transcripts. Restriction enzyme digestion revealed advantage over physical methods for cDNA fragmentation, such as prompt identification of spurious gene fusion reads produced during cDNA library construction, by the presence of enzymatic restriction sites in the fusion junction of the reads.

High-throughput transcriptome sequencing has been used to identify genomic rearrangements resulting in gene fusion events [23], [35], [36], [37] and superior sensitivity was achieved when paired-end sequencing was applied [36], [37], [38]. Single-end sequencing of long or short reads has led to low validation rates, which seem to be increased when single-end long and short sequencing are used in combination [35]. The low validation rate obtained here (21%) reinforces the difficulty of confirming these events using PCR-based approaches. Nonetheless, despite using single-end long reads sequencing we identified three bona fide gene fusions, reported here for the first time to the best of our knowledge.

One of the validated gene fusions reported 10 nt of micro-homology sequence, also detected in 82% of the gene fusion candidates, probably resulting from a replication mechanism known as Fork Stalling and Template Swichting Model (FoSTeS). These replication disorders arises due to nucleotide similarities between DNA strands [39], and have been detected in breast cancer samples [40].

For exploring SNPs, we used stringent bioinformatics and manual inspection that resulted in a high rate of confirmation (89%), including validated SNPs with apparent allelic dosage imbalance in C5.2 cells. Whether *ERBB2* overexpression can mediate allelic dosage imbalance during transcriptional process remains to be addressed.

The detection of alternative splicing by our method is enhanced by the longer fragments produced by the 454-platform, compared to other next-generation sequencing technologies. Here we showed a 90% validation rate of the exon inclusion splicing variant class. An extrapolation of this value over the 1,704 novel AS events in multi-exon splicing variants, with conserved splice sites, identified by RNAseq would result in 1,533 bona-fide AS events. Our approach therefore demonstrates a high capacity for identification of novel splicing variants and, consequently, for the definition of the mammary transcriptome.

Amplification of the *ERBB2* oncogene is considered an important tumor driver [41], and has been reported in approximately 25% of breast cancers [11]. The quantitative transcriptional aspect of overexpression of the oncogene has been previously assessed by 3′ end sequence methodology [18], [19]. However, only whole transcriptome sequencing enables the assessment of some relevant structural aspects. The influence of *ERBB2* was observed in quantitative aspects of breast cell line transcriptomes, not only on gene expression but also on specific splicing variants. Enrichment of exon skipping/inclusion and alternative splice site selection by *ERBB2* overexpression in C5.2 cells, observed in the current study, indicated a potential influence of the oncogene in the regulation of the splicing process. In this sense, evidence has already been presented by us concerning expression level regulation of specific AS variants mediated by *ERBB2* [42]. Additionally, it has been suggested by others [43] that activation of signaling pathways such as Ras/MAPK and PI3K/AKT, which are controlled in part by ERBB2 signaling, might influence the alternative splicing balance of cells, by phosphorylation and activation of specific splicing factors.

The intrinsic molecular heterogeneity found between distinct human tumor samples as well as within a single breast tumor sample has been reported by many laboratories [7], [44]. These differences appear to be strongly dependent upon microenvironmental factors [5], [45]. Despite differences in molecular characteristics between cells *in vivo* and *in vitro*, our approach allowed us to identify 4 genes, the expression of which is likely mediated by *ERBB2*. We highlight *LOX* downregulation in C5.2 cells as well as in tumor samples overexpressing *ERBB2*. Furthermore, our data also revealed a higher level of *LOX* expression in C5.2 cells after exposure to rapamycin (4-fold change), data indicating that *LOX* is potentially regulated by the ERBB2/mTOR pathway. *LOX* encodes an extracellular copper-requiring enzyme that initiates collagen and elastin crosslinking and enhances tumor cell invasion and metastasis [46]. Conversely, the 18-kDa LOX propeptide was found to be an effective inhibitor of the more invasive phenotype of breast cancer cells driven by *ERBB2* and has been suggested to improve treatment in this subtype of breast cancer [47].

Altogether, the results presented here demonstrate that our approach is suitable for whole transcriptome interrogation, with single or multiple samples in parallel sequencing by the 454-ROCHE platform, from which an accurate quantitative and qualitative portrait of complex transcriptomes can be generated.

## Materials and Methods

### Cell lines and tumor samples: treatment and RNA purification

Two human breast cell lines, HB4a [16] and C5.2 [17], were provided by Dr. Michael O'Hare – LICR, NY, and were grown as described [16]. The high expression of ERBB2 transcript in C5.2 compared to HB4a was confirmed by quantitative RT-PCR (data not shown). For rapamycin treatment, both cell lines were plated in 25 cm^2^ flasks and maintained at 40–50% confluence. Cells were treated with vehicle (0.01% absolute ethanol) or 20 nM rapamycin for 24 hours.

Breast tumor samples containing signed informed consents were retrieved from the A. C. Camargo Hospital Biobank. This study is in accordance with the Ethics Committee in Human Being Research from Fundação Antonio Prudente – Hospital of Cancer - A.C. Camargo/S.P and has been approved under number 952/07. Fresh-frozen tumor blocks were cut, fixed, and stained with hematoxylin and eosin (H&E) and reviewed by a pathologist. Total RNA was extracted with Trizol and treated with DNaseI. Samples were classified as ERBB2-high or basal expression according to protein and mRNA levels. Protein levels were evaluated by immunohistochemistry (signals 2+ or 3+ indicating high ERBB2 expression, and signals 0 or 1+ indicating basal expression). ERBB2 transcript was evaluated by quantitative RT-PCR. Only samples with concordant results at both protein and transcript expression level were included in the study.

### Whole-transcriptome libraries and Roche-platform sequencing

Two hundred nanograms of mRNA poly A^+^ was incubated with 0.5 µg oligo-dT containing a *Dpn*II restriction site [5′GAGGCGGGATCT(30)3′]. First and second strand cDNA synthesis were carried out as described [11]. Purified dscDNA was digested with 25 units of *Dpn*II at 37°C for 3 hours. Next, Y-shaped DNA adapters [48] were added to dscDNA fragments with T4 DNA ligase at 16°C overnight. The HB4a and C5.2 Y-shaped adapters were formed by primers A and B and primers C and D, respectively (Primer A: 5′-GATCTCCCGAGTGGTCACCTGCTC-3′; Primer B: 5′-CTAGCAGCTACCACTCGGGA-3′; Primer C: 5′-GATCCCCTGAGTGGTCACCTGCTC-3′, and Primer D: 5′-CTAGCAGCTACCACTCAGGG-3′). Fragments ranging from 150 bp to 600 bp were size-selected by agarose gel electrophoresisPurified products were amplified in a 20-cycle PCR, with 2 units Platinum *Taq* DNA Polymerase High-Fidelity (Invitrogen, Carlsbad, California), 0.2 mM dNTPs, 2 mM MgCl_2_, and 5 pmol each of forward (5′GAGCAGGTGACCACTC3′) and reverse (5′CTAGCAGCTACCACTC3′) primers. PCR products were verified in 1% ethidium-bromide-agarose gels. Equivalent masses of each cell line (HB4a and C5.2) were pooled, and 1.5 µg of the cDNAs from the poly A^+^ library were submitted to Titanium Genome Sequencer FLX System 454 Roche-Life Sciences sequencing.

### Bioinformatics analyses

454-Roche Titanium reads were screened for the presence of human ribosomal RNA or mitochondrial DNA by MEGABLAST (E-value≤1×10^−20^, identity ≥85% and coverage ≥90%). Next, reads were aligned against the human genome (release hg18, March 2006) through BLAT [49]. Completely aligning reads were defined as reads aligning with coverage ≥70% and identity ≥96% (pslReps parameters: minCover = 0.70, minAli = 0.96, nearTop = 0.005). Reads with significant hits at multiple genome locations were discarded.

For gene fusion events, reads from partly aligning subset, defined as reads with coverage ≥20% and ≤80% and identity ≥99.9% (pslReps parameters: minCover = 0.70, minAli = 0.96, nearTop = 0.005) were used. Next each region of the read could have only one single match in the human genome sequence and the two regions of the read should align to different genes. Chimeric events including one gene and its corresponding pseudogene were also discarded. Additionally, only reads mapping to exonic regions were selected.

To determine the sequence depth coverage of the fusion gene candidates, we performed alignments between the read reporting the fusion event and all reads generated by 454-sequencing using less stringent criteria.

The KnownGene [50] annotation track coordinates from the University of California Santa Cruz genome database (UCSC) was used as a reference for mapping of the reads in relation to annotated transcripts and exons. Gaps spanning more than 50 bp and having conserved splice sites were considered true intron sequences, and were used to identify putative splicing events not annotated in the KnownGene annotation track. Reads not mapped to any gene-track were used for the identification of putative novel genes. These candidates were analyzed for the presence of conserved splice sites.

For single nucleotide polymorphisms (SNPs) Blast-like BLAT alignment outputs were parsed by the use of a Perl script. A set of parameters such as base coverage, proximity to exon-intron boundaries, proximity to alignment ends, number of different sequenced bases for a specific genome location, ratio between divergent base and reference base, and presence in both libraries, was used to select putative SNPs, that were aligned against dbSNP (build 129) [51].

For all comparative analyses of both RNA-seq from HB4 and C5.2 cells, DNA barcoding identification was valid when barcoding was flanked by a 5′-adapter sequence and a 3′-restriction enzyme site. To analyze the differential gene expression profile between HB4a and C5.2 cell lines, we first aligned confident reads against the RefSeq database with the MegaBlast tool (E-value≤1×10−15, identity ≥96% and coverage ≥90%). Reads with significant alignments to different genes were excluded. The overall read count *per* gene was scaled to reads per million (RPM), and differential expression was calculated as the ratio of C5.2RPM/HB4aRPM. We used the SAGEbetaBin statistical approach to calculate the Bayes Error Rate (SAGEbetaBin available at http://bioinfo.lbhc.hcancer.org.br/sage/betabin//en/index.php) [52].

Association between RNA-Seq read count and qRT_PCR Ct-values was assessed with Spearman correlation coefficient and p<0.01 was considered as statistically significant.

### Validation of SNPs

Potentially novel SNPs exhibiting more than one mismatch in a 50 bp window or mapped to homopolymers and repetitive sequences were discarded. Primers were designed by Primer3 and were used for PCR amplification with DNA from HB4 and C5.2 cell lines. The amplicons were evaluated on 3% agarose gels and were sequenced in an ABI 3130xL (Applied Biosystems, Foster City, California).

### Validation of gene fusions

For confirmation of gene fusion, two strategies were used. First, primers aligning at the extremities of the reads reporting the event were designed for RT-PCR and PCR amplification using both cDNA and genomic DNA, respectively, from both cell lines (Table S7). Amplification products were analyzed on 8% polyacrylamide gels and were sequenced on the ABI3130 instrument (Applied Biosystems, Foster City, California).

Further, for detection of fusion events in cDNA two probes (left and right) were designed for each putative gene fusion event. The left probe was complementary to one of the genes involved in the event, exactly at the limit of the fusion; the right probe, directly adjacent to the left probe, was complementary to the other gene involved in the putative event. In addition, the left probe contained at its 5′ end a recognition sequence of the forward PCR primer (5′GGGTAGGCTAAGGGTAGGA3′). The right probe was phosphorylated at its 5′ end and contained a recognition sequence of the reverse PCR primer (5′TCTAGATTGGATCTTGCTGGCAC3′) at its 3′ end (Table S7). The probes were hybridized to pre-heated double-stranded cDNA and genomic DNA from HB4a and C5.2 cells at 54°C for 12 hours. The two probes hybridized to their target sequence were subsequently ligated by Ligase-65 (MC Holland, Amsterdam, Netherlands) to form a single probe that was PCR-amplified. As a negative control, hybridization in the absence of any template was performed for all probes, and the reaction was submitted to PCR. Amplification products were analyzed on 8% polyacrylamide gels and were sequenced on the ABI3130 instrument (Applied Biosystems, Foster City, California).

### Validation of splicing variants

Primers were designed at the respective novel exon and at one adjacent exon (Table S8). cDNAs converted from 40 ng of DNase I-treated total RNA from HB4a and C5.2 cells was used in each reaction. PCR was performed in a total volume of 20 µl, 1 X reaction buffer, 2.5 mM MgCl_2_, 0.2 mM dNTP, 10 pmoles of each primer, and 1 unit Platinum Taq DNA polymerase (Life Technologies, Foster City, California) in 40 cycles at 95°C for 30 sec, 60°C for 30 sec, and 72°C for 30 sec, followed by a final extension at 72°C for 7 min. Amplification products were visualized on 8% polyacrylamide gels and were sequenced on an ABI3130 instrument (Applied Biosystems Foster City, California). For quantitative analyses, PCR amplification with the same pair of primers was performed in 20 µl 1 X SYBR Green PCR MasterMix (Applied Biosystems Foster City, California), containing 2–8 pmoles of each primer and cDNA converted from 100 ng of total RNA.

### Validation of novel genes

Primers for the validation of 6 putative novel genes were designed at two distinct exons with the 454-read as a reference sequence (Table S9). cDNA converted from 40 ng of DNase I-treated total RNA from HB4a and C5.2 cells was used in each reaction. PCR reactions were performed in 20 µl containing 1 X buffer, 2.5 mM MgCl_2_, 0.2 mM dNTP, 10 pmoles of each primer, and 1 unit Taq DNA polymerase incubated at 95°C for 30 sec, 60°C for 30 sec, and 72°C for 30 sec for 40 cycles, followed by a final extension at 72°C for 7 min. Amplification products were visualized on 8% polyacrylamide gels and were sequenced on an ABI3130 instrument (Applied Biosystems Foster City, California).

### Validation of differential gene expression

cDNA converted from 400 ng of total RNA was used as a template for the evaluation of 96 distinct transcripts (target genes and endogenous controls) in duplicate. Expression levels of selected genes were verified by quantitative RT-PCR with customized TaqMan low-density arrays (Applied Biosystems Foster City, California) in an ABI7900 instrument. A total of 91 target genes (75 and 16 up-regulated genes in C5.2 and HB4a, respectively) was randomly selected; *GUSB* was selected, from the 5 endogenous genes tested, as a reference gene. Differential expression levels that exhibited a fold-change>2, were considered significant as determined by the 2^−ΔΔCt^ method. The list of selected genes is shown in Table S5.

### Gene Ontology (GO) and KEGG pathways annotation

FunNet tools were used for computation of the enriched GO and KEGG categories [29]. Significant themes were calculated for up- and down-regulated genes, with the 11,366 represented genes as the reference set. A decorrelated annotation procedure was performed by application of the Fisher exact test using corrected *p*-values (*p*-value<0,01) and false discovery rate 5%.

## Supporting Information

Figure S1 **Relative position frequency to RefSeq transcripts.** (A) The frequency of reads distributed along transcript position from the Poly (A)^+^ library, where 0 is the 5′end and 100 is the 3′end of each corresponding transcript. (B) The relative transcript position of sequences from the Poly (A)^+^ library in relation to transcript size. The thickness of bars corresponds to the frequency of sequences in each group.
(TIF)

Figure S2 **Validation of novel SNPs.** The chromatogram represents the validation of the SNPs for each gene. The SNPs from the HB4a and C5.2 cell lines are shown separately and are classified as homozygous or heterozygous.
(TIF)

Figure S3 **Validation of alternative splicing variants by RT-PCR.** Each validated AS event is represented by the genomic coordinates of each exon/intron border. The blank squares represent the constitutive exons and the grey squares represent the alternative exons. The gene symbol and corresponding RefSeq entry used as a reference are also shown. qRT-PCR validation: The 8 genes evaluated by RT-PCR are separated by the double line, and the results are shown inside the square as up- or down-regulation in the corresponding cell lines.
(TIF)

Figure S4 **Identification of artefactual chimeric transcripts.** Reads containing *Dpn*II restriction site at the border junction of chimeric transcripts were discarded from gene fusion analysis. In this example, a chimeric read between the genes *CAPNS1* (chromosome 19) and *FAM156A* (chromosome X) is shown. Colored lines highlight the regions involved in the fusion. The nucleotide sequence corresponding to each gene is shown with the *Dpn*II restriction site highlighted in larger font size (GATC).
(TIF)

Figure S5 **Classification of differently expressed genes according to Gene Ontology and Kegg Pathways.** GO (Biological Process) and KEGG enriched categories in the 46 differentially expressed genes validated. The bar corresponds to the percentage of differentially expressed genes in relation to all annotated genes of the RNA-seq in the respective category. Up- and down-regulated genes refer to the C5.2 cell line.
(TIF)

Table S1 **Validation of SNPs.** The gene symbol is used to identify each selected SNP. The SNPs are localized according to untranslated region (5′ and 3′ UTR) or coding sequence (CDS) and mRNA coordinates. The nucleotide alteration is shown. The amino acid alteration is shown only for non-synonymous cases. Genotype identified for HB4a and C5.2 cell lines after Sanger sequencing is shown. ND: Not determined.
(DOC)

Table S2 **Characterization of gene fusion events.** Gene symbol of the genes involved in each fusion event are given. The total number of reads reporting the fusion event is shown and also the number reads identified by each library is shown in parenthesis (C – C5.2 cells; H – Hb4a cells; U – reads of undefined origin). MH – Number of bases with microhomology between the genes. NT – Number of bases of short non-templated sequences. Genes involved in more than one event identified by our data are colored red. Genes involved in fusion events reported in the literature are shown, and the corresponding genes(s) reported are identified by their gene symbol. Light green highlights the events selected for validation. Bright green highlights validated gene fusions.
(DOC)

Table S3 **Alternative splicing events detected for each cell line.** The number of alternative splicing events detected for each sample normalized by the total number of reads generated for each cell line.
(DOC)

Table S4 **Alternative splicing variants modulated by ERBB2 expression.** The position of the novel exon identified is shown according to the number of the flanking exons. The expression level obtained by qRT-PCR is reported as fold-change between C5.2 and HB4a.
(DOC)

Table S5 **Validation of differential gene expression modulated by** ***ERBB2*** **.** The mRNA seq data is given as a fold-change between C5.2 and HB4a cell lines. When no reads were identified in the RNA-seq from one of the cell lines, we calculated fold-change by replacing “0” by “1”. Positive and negative values correspond to higher expression in C5.2 and HB4a, respectively. The qRT_PCR results are given as fold-change obtained by 2^−ΔΔCT^. In grey: genes validated in the qRT-PCR by the criterion for differentially expressed genes as Fold-change>2.
(DOC)

Table S6 **Effects of rapamycin treatment on genes influenced by** ***ERBB2*** **-mediated expression.** The results from quantitative RT-PCR on rapamycin-treated cell lines for the 46 validated genes are given as fold-change between C5.2 and HB4a cell lines. Positive and negative values correspond to higher expression in C5.2 and HB4a cell lines, respectively. The fourth columns show the qRT_PCR results obtained from the C5.2 and HB4a cell lines and from the cells lines after rapamycin treatment. Response to rapamycin was considered when a decrease or inversion of fold-change between C5.2 and HB4a cell lines compared to non-treated cell lines was observed. Yes and No represent response or no response to rapamycin, respectively. (nd) Cycle threshold not determined.
(DOC)

Table S7 **Validation of gene Fusion.** The gene fusions evaluated are characterized by the 2 chromosomes involved in the event as well as the corresponding genes. The specific hybridization sequence of the probes and the amplicon size expected after PCR are shown.
(DOC)

Table S8 **Novel alternative splicing variants.** The forward and reverse primer sequences used for each validation are shown with the corresponding gene symbol and the amplicon size.
(DOC)

Table S9 **Putative novel genes.** The chromosome localization of each putative novel gene is shown as well as the sequences of forward and reverse primers and the respective amplicon size.
(DOC)

## References

[1] Veer LJ, Dai H, van de Vijver MJ, He YD, Hart AA (2002). Gene expression proffiling predicts clinical outcome of breast cancer.. Nature.

[2] Brentani RR, Carraro DM, Verjovski-Almeida S, Reis EM, Neves EJ (2005). Gene expression arrays in cancer research: methods and applications.. Crit Rev Oncol Hematol.

[3] Folgueira MA, Carraro DM, Brentani H, Patrão DF, Barbosa EM (2005). Gene expression profile associated with response to doxorubicin-based therapy in breast cancer.. Clin Cancer Res.

[4] Castro NP, Osório CA, Torres C, Bastos EP, Mourão-Neto M (2008). Evidence that molecular changes in cells occur before morphological alterations during the progression of breast ductal carcinoma.. Breast Cancer Res.

[5] Rozenchan PB, Carraro DM, Brentani H, de Carvalho Mota LD, Bastos EP (2009). Reciprocal changes in gene expression profiles of cocultured breast epithelial cells and primary fibroblasts.. I J Cancer.

[6] Koike FMA, Brentani H, Carraro DM, De Camargo BFM, Hirata KML (2009). Gene expression profile of residual breast cancer after doxorubicin and cyclophosphamide neoadjuvant chemotherapy.. Oncol Rep.

[7] Perou CM, Sørlie T, Eisen MB, van de Rijn M, Jeffrey SS (2000). Molecular portraits of human breast tumours.. Nature.

[8] Sørlie T, Perou CM, Tibshirani R, Aas T, Geisler S (2001). Gene expression patterns of breast carcinomas distinguish tumor subclasses with clinical implications.. Proc Natl Acad Sci U S A.

[9] Ross JS, Fletcher JA (1998). The HER-2/neu Oncogene in Breast Cancer: Prognostic Factor, Predictive Factor, and Target for Therapy.. Oncologist.

[10] Campbell CI, Petrik JJ, Moorehead RA (2010). ErbB2 enhances mammary tumorigenesis, oncogene-independent recurrence and metastasis in a model of IGF-IR-mediated mammary tumorigenesis.. Molecular Cancer.

[11] Slamon DJ, Godolphin W, Jones LA, Holt JA, Wong SG (1989). Studies of the HER-2/neu proto-oncogene in human breast and ovarian cancer.. Science.

[12] Slamon DJ, Clark GM, Wong SG, Levin WJ, Ullrich A (1987). Human breast cancer: correlation of relapse and survival with amplification of the HER-2/neu oncogene.. Science.

[13] Purdie CA, Baker L, Ashfield A, Chatterjee S, Jordan LB (2010). Increased mortality in HER2 positive, oestrogen receptor positiveinvasive breast cancer: a population-based study.. B J Cancer.

[14] Ménard S, Pupa SM, Campiglio M, Tagliabue E (2003). Biologic and therapeutic role of HER2 in cancer.. Oncogene.

[15] Vogel CL, Cobleigh MA, Tripathy D, Gutheil JC, Harris LN (2002). Efficacy and Safety of Trastuzumab as a Single Agent in First-Line Treatment of HER2-Overexpressing Metastatic Breast Cancer.. Journal of Clinical Oncology.

[16] Stamps AC, Davies SC, Burman J, O'Hare MJ (1994). Analysis of proviral integration in human mammary epithelial cell lines immortalized by retroviral infection with a temperature-sensitive SV40 T-antigen construct.. I J Cancer.

[17] Harris RA, Eichholtz TJ, Hiles ID, Page MJ, O'Hare MJ (1999). New model of ErbB-2 over-expression in human mammary luminal epithelial cells.. Int J Cancer.

[18] Dos Santos ML, Palanch CG, Salaorni S, Da Silva WA, Nagai MA (2006). Transcriptome characterization of human mammary cell lines expressing different levels of ERBB2 by serial analysis of gene expression.. Int J Oncol.

[19] Dos Santos ML, Gimenes KP, Silva WA, Nagai MA (2009). Transcriptome changes induced by docetaxel in human mammary cell lines expressing different levels of ERBB2.. Int J Mol Med.

[20] Graveley BR (2001). Alternative splicing: increasing diversity in the proteomic world.. Trends Genet.

[21] Harrow J, Denoeud F, Frankish A, Reymond A, Chen CK (2006). GENCODE: producing a reference annotation for ENCODE.. Genome Biol.

[22] Torres TT, Metta M, Ottenwälder B, Schlötterer C (2008). Gene expression profiling by massively parallel sequencing.. Genome Res.

[23] Maher CA, Palanisamy N, Brenner JC, Cao X, Kalyana-Sundaram S (2009). Chimeric transcript discovery by paired-end transcriptome sequencing.. Proc Nat Acad Sci U S A.

[24] Tang F, Barbacioru C, Wang Y, Nordman E, Lee C (2009). mRNA-Seq whole-transcriptome analysis of a single cell.. Nature Methods.

[25] Yassour M, Kaplan T, Fraser HB, Levin JZ, Pfiffner J (2009). Ab initio construction of a eukaryotic transcriptome by massively parallel mRNA sequencing.. Proc Natl Acad Sci U S A.

[26] Pleasance ED, Stephens PJ, O'Meara S, McBride DJ, Meynert A (2010). A small-cell lung cancer genome with complex signatures of tobacco exposure.. Nature.

[27] Dias-Neto E, Correa RG, Verjovski-Almeida S, Briones MR, Nagai MA (2000). Shotgun sequencing of the human transcriptome with ORF expressed sequence tags.. Proc Natl Acad Sci U S A.

[28] Ramensky V, Bork P, Sunyaev S (2002). Human non-synonymous SNPs: server and survey.. Nucleic Acids Res.

[29] Prifti E, Zucker J, Clement K, Henegar C (2008). FunNet: an integrative tool for exploring transcriptional interactions.. Bioinformatics.

[30] Janus A, Robak T, Smolewski P (2005). The mammalian target of the rapamycin (mTOR) kinase pathway: its role in tumourigenesis and targeted antitumour therapy.. Cell Mol Biol Lett.

[31] Creighton CJ (2007). A gene transcription signature of the Akt/mTOR pathway in clinical breast tumors.. Oncogene.

[32] Heinonen H, Nieminen A, Saarela M, Kallioniemi A, Klefström J (2008). Deciphering downstream gene targets of PI3K/mTOR/p70S6K pathway in breast cancer.. BMC Genomics.

[33] Akcakanat A, Zhang L, Tsavachidis S, Meric-Bernstam F (2009). The rapamycin-regulated gene expression signature determines prognosis for breast cancer.. Mol Cancer.

[34] Meric-Bernstam F, Gonzalez-Angulo AM (2009). Targeting the mTOR signaling network for cancer therapy.. J Clin Oncol.

[35] Maher CA, Kumar-Sinha C, Cao X, Kalyana-Sundaram S, Han B (2009). Transcriptome sequencing to detect gene fusions in cancer.. Nature.

[36] Edgren H, Murumagi A, Kangaspeska S, Nicorici D, Hongisto V (2011). Identification of fusion genes in breast cancer by paired-end RNA-sequencing.. Genome Biol.

[37] McManus CJ, Duff MO, Eipper-Mains J, Graveley BR (2010). Global analysis of trans-splicing in Drosophila.. Proc Natl Acad Sci U S A.

[38] Wang XS, Prensner JR, Chen G, Cao Q, Han B (2009). An integrative approach to reveal driver gene fusions from paired-end sequencing data in cancer.. Nat Biotechnol.

[39] Gu W, Zhang F, Lupski JR (2008). Mechanisms for human genomic rearrangements.. Pathogenetics.

[40] Stephens PJ, McBride DJ, Lin ML, Varela I, Pleasance ED (2009). Complex landscapes of somatic rearrangement in human breast cancer genomes.. Nature.

[41] Di Fiore PP, Pierce JH, Kraus MH, Segatro O, King CR (1987). Erbb-2 is a potent oncogene when overexpressed in NIHI/3T3 Cells.. Science.

[42] Ferreira EN, Rangel MC, Galante PF, de Souza JE, Molina GC (2010). Alternative splicing enriched cDNA libraries identify breast cancer-associated transcripts.. BMC Genomics.

[43] Srebrow A, Kornblihtt AR (2006). The connection between splicing and cancer.. J Cell Sci.

[44] Stingl J, Caldas C (2007). Molecular heterogeneity of breast carcinomas and the cancer stem cell hypothesis.. Nat Rev Cancer.

[45] Allinen M, Beroukhim R, Cai L, Brennan C, Lahti-Domenici J (2004). Molecular characterization of the tumor microenvironment in breast cancer.. Cancer Cell.

[46] Noblesse E, Cenizo V, Bouez C, Borel A, Gleyzal C (2004). Lysyl oxidase-like and lysyl oxidase are present in the dermis and epidermis of a skin equivalent and in human skin and are associated to elastic fibers.. J Invest Dermatol.

[47] Min C, Kirsch KH, Zhao Y, Jeay S, Palamakumbura AH (2007). The tumor suppressor activity of the lysyl oxidase propeptide reverses the invasive phenotype of Her-2/neu-driven breast cancer.. Cancer Res.

[48] Watahiki A, Waki K, Hayatsu N, Shiraki T, Kondo S (2004). Libraries enriched for alternatively spliced exons reveal splicing patterns in melanocytes and melanomas.. Nat Methods.

[49] Kent WJ (2002). BLAT–the BLAST-like alignment tool.. Genome Res.

[50] Hsu F, Kent WJ, Clawson H, Kuhn RM, Diekhans M (2006). The UCSC Known Genes.. Bioinformatics.

[51] Sherry ST, Ward MH, Kholodov M, Baker J, Phan L (2001). dbSNP: the NCBI database of genetic variation.. Nucleic Acids Res.

[52] Vêncio RZ, Brentani H, Patrão DF, Pereira CA (2004). Bayesian model accounting for within-class biological variability in Serial Analysis of Gene Expression (SAGE).. BMC Bioinformatics.

