# Correction: Poly (A)^+^ Transcriptome Assessment of *ERBB2*-Induced Alterations in Breast Cell Lines

**DOI:** 10.1371/annotation/b8888ae6-e402-43af-a425-6fd5e58deef6

**Published:** 2011-07-26

**Authors:** Dirce Maria Carraro, Elisa Napolitano Ferreira, Gustavo de Campos Molina, Renato David Puga, Eduardo Fernandes Abrantes, Adriana Priscila Trapé, Bedrich L. Ekhardt, Diana Noronha Nunes, Maria Mitzi Brentani, Wadih Arap, Renata Pasqualini, Helena Brentani, Emmanuel Dias-Neto, Ricardo Renzo Brentani

The 7th author's name is misspelled. The correct spelling is: Bedrich L. Eckhardt 

