# Supplementary figures and images for: Poly (A)+ Transcriptome Assessment of ERBB2-Induced Alterations in Breast Cell Lines
Source: PLoS One. 2011 Jun 22;6(6):e21022. doi: 10.1371/journal.pone.0021022 (PMC3120832; doi:10.1371/journal.pone.0021022)

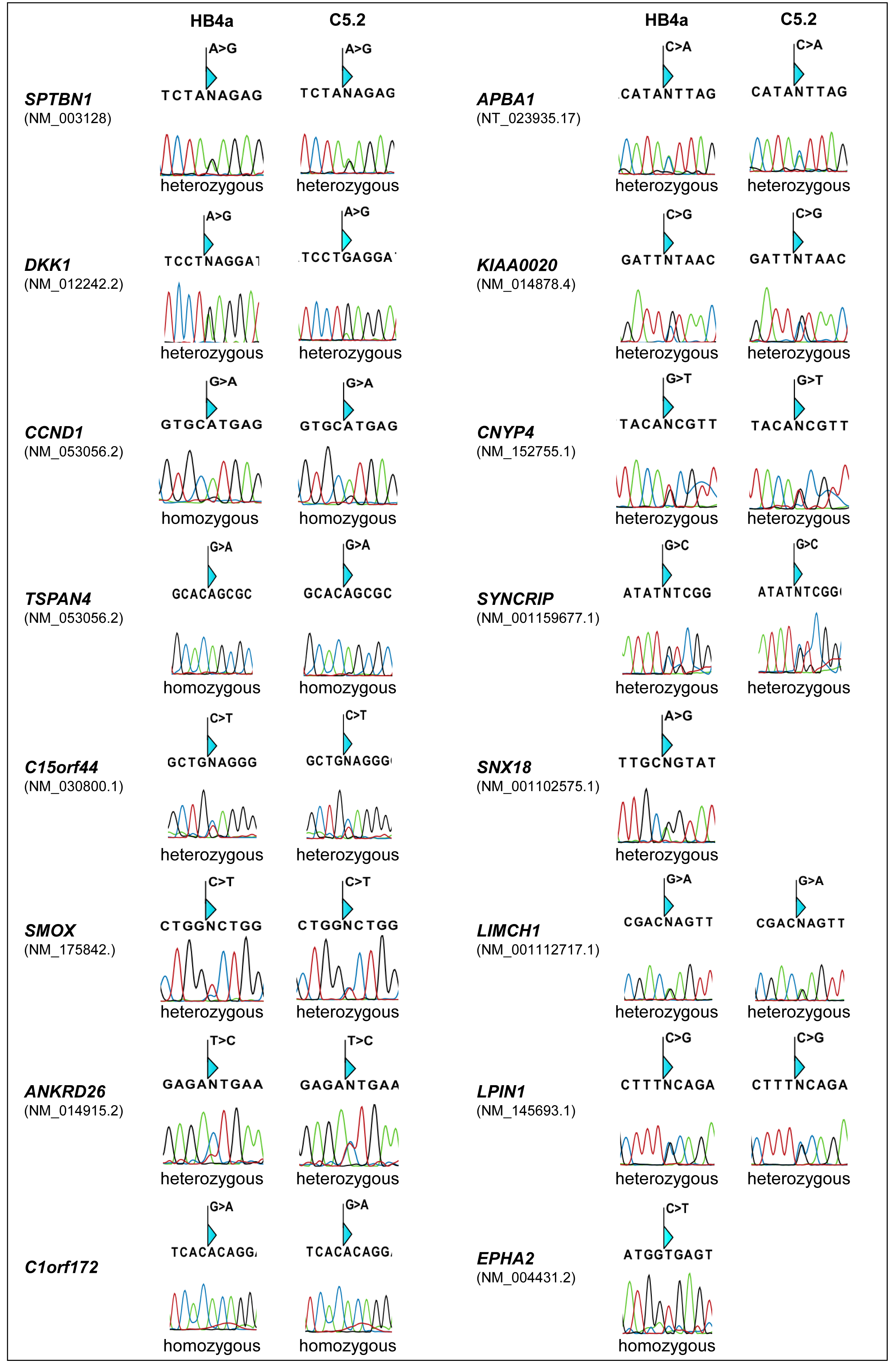

Supplement: Figure S2 — Validation of novel SNPs. The chromatogram represents the validation of the SNPs for each gene. The SNPs from the HB4a and C5.2 cell lines are shown separately and are classified as homozygous or heterozygous. (TIF) [file pone.0021022.s002.tif]

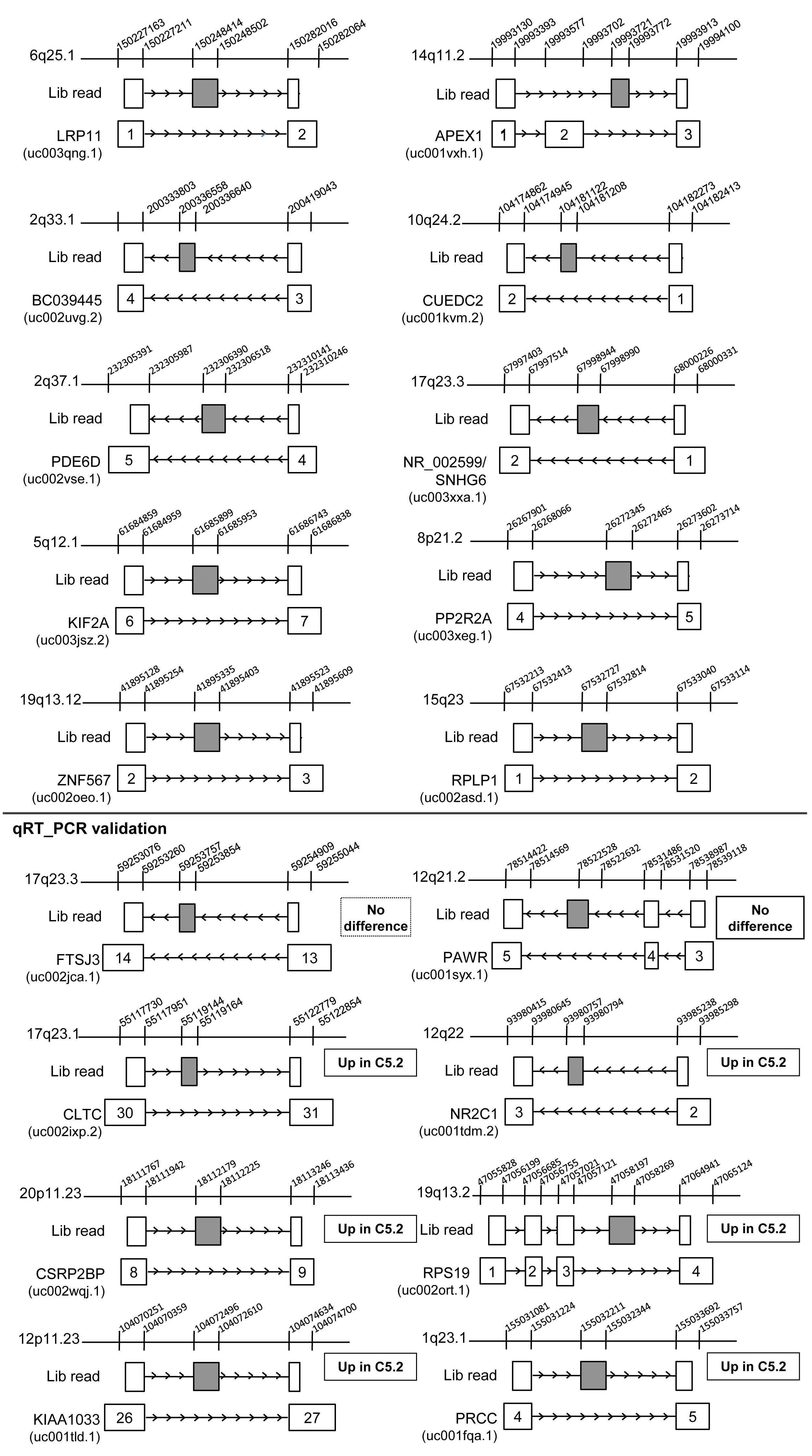

Supplement: Figure S3 — Validation of alternative splicing variants by RT-PCR. Each validated AS event is represented by the genomic coordinates of each exon/intron border. The blank squares represent the constitutive exons and the grey squares represent the alternative exons. The gene symbol and corresponding RefSeq entry used as a reference are also shown. qRT-PCR validation: The 8 genes evaluated by RT-PCR are separated by the double line, and the results are shown inside the square as up- or down-regulation in the corresponding cell lines. (TIF) [file pone.0021022.s003.tif]

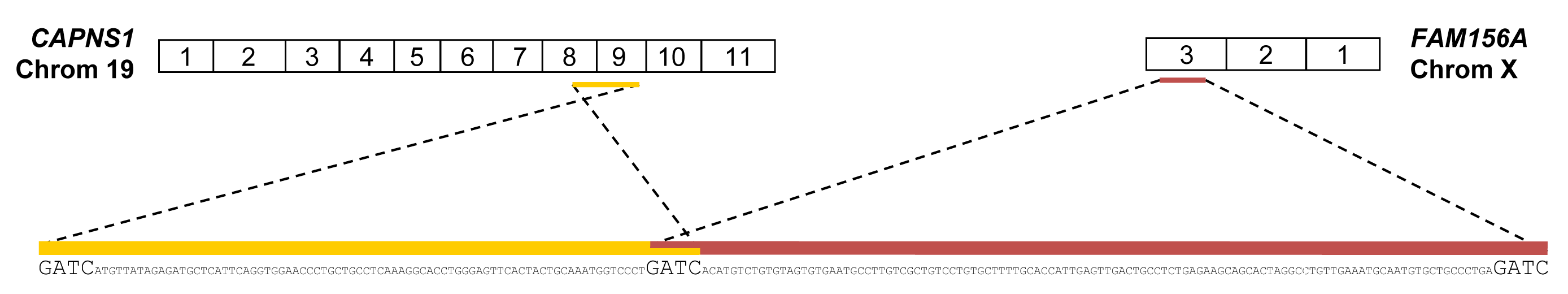

Supplement: Figure S4 — Identification of artefactual chimeric transcripts. Reads containing DpnII restriction site at the border junction of chimeric transcripts were discarded from gene fusion analysis. In this example, a chimeric read between the genes CAPNS1 (chromosome 19) and FAM156A (chromosome X) is shown. Colored lines highlight the regions involved in the fusion. The nucleotide sequence corresponding to each gene is shown with the DpnII restriction site highlighted in larger font size (GATC). (TIF) [file pone.0021022.s004.tif]

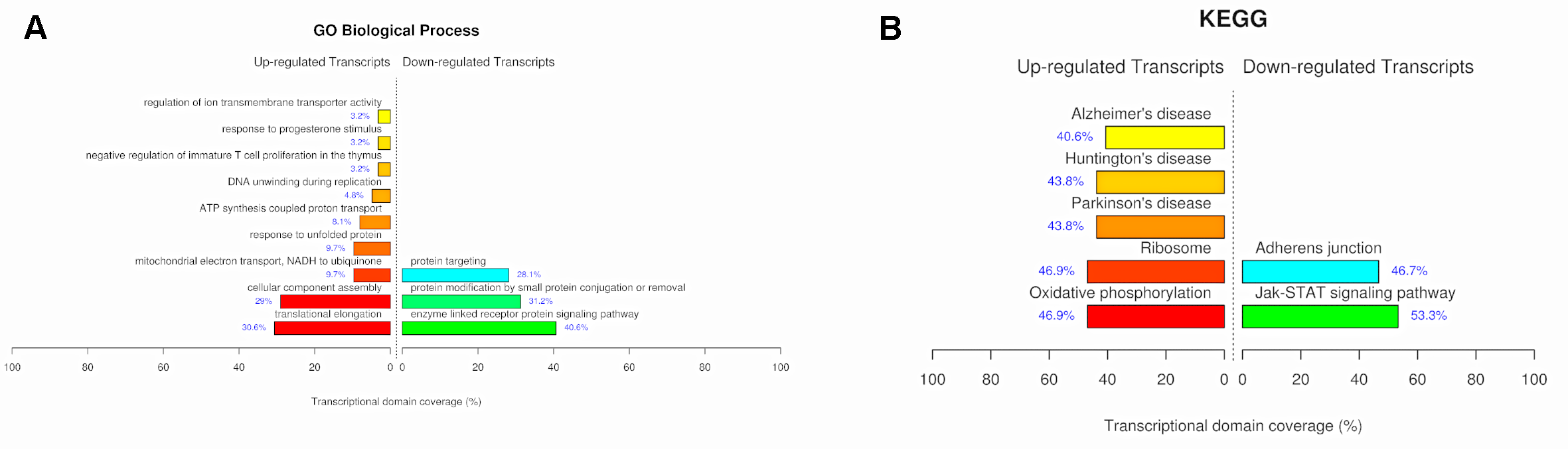

Supplement: Figure S5 — Classification of differently expressed genes according to Gene Ontology and Kegg Pathways. GO (Biological Process) and KEGG enriched categories in the 46 differentially expressed genes validated. The bar corresponds to the percentage of differentially expressed genes in relation to all annotated genes of the RNA-seq in the respective category. Up- and down-regulated genes refer to the C5.2 cell line. (TIF) [file pone.0021022.s005.tif]
