# Supplementary material for: Poly (A)+ Transcriptome Assessment of ERBB2-Induced Alterations in Breast Cell Lines
Source: PLoS One. 2011 Jun 22;6(6):e21022. doi: 10.1371/journal.pone.0021022 (PMC3120832; doi:10.1371/journal.pone.0021022)
Supplement: Table S1 — Validation of SNPs. The gene symbol is used to identify each selected SNP. The SNPs are localized according to untranslated region (5′ and 3′ UTR) or coding sequence (CDS) and mRNA coordinates. The nucleotide alteration is shown. The amino acid alteration is shown only for non-synonymous cases. Genotype identified for HB4a and C5.2 cell lines after Sanger sequencing is shown. ND: Not determined. (DOC) [file pone.0021022.s006.doc]

| Gene Symbol | mRNA location | | Alteration | Sanger Sequencing | |
| --- | --- | --- | --- | --- | --- |
|  | region/position | EXON | nts (aa) | Validation in HB4 | Validation in C5.2 |
| ANKRD26 | CDS/4617 | 30 | T>C (I>T) | T/C | T/C |
| APBA1 | 3’ UTR/6186 | 13 | C>A | C/A | C/A |
| C15ORF44 | CDS/1524 | 10 | C>T | C/T | C/T |
| C1ORF172 | CDS/942 | 2 | G>A | A/A | A/A |
| CCND1 | 3’ UTR/3430 | 4 | G>A | A/A? | A/A? |
| CNYP4 | CDS/342 | 2 | G>T (S>I) | G/T | G/T |
| DKK1 | 3’ UTR/1037 | 4 | A>G | G/A | G/G |
| EPHA2 | CDS/2264 | 13 | C>T | T/T | NA |
| EXOC6 | CDS/1169 | 7 | C>A (A>D) | C/C | C/C |
| KIAA0020 | CDS/1036 | 9 | C>G (L>V) | C/G | C/G |
| LIMCH1 | 3’ UTR/4467 | 26 | G>A | G/A | G/A |
| LPIN1 | 3’ UTR/4826 | 20 | C>G | C/G | C/G |
| SMOX | CDS/108 | 2 | C>T | C/T | C/T |
| SNX18 | 3’ UTR/4387 | 2 | A>G | A/G | NA |
| SPG11 | CDS/5587 | 30 | A>G | A/A | A/A |
| SPTBN1 | CDS/6734 | 33 | A>G (K>R) | A/G | A/G |
| SYNCRIP | 3’ UTR/2309 | 11 | T>G | T/G | T/G |
| TBC1D9B | 3’ UTR/4132 | 22 | C>A | N/A | N/A |
| TSPAN4 | CDS/765 | 7 | G>A | A/A | A/A |
