# Supplementary material for: Poly (A)+ Transcriptome Assessment of ERBB2-Induced Alterations in Breast Cell Lines
Source: PLoS One. 2011 Jun 22;6(6):e21022. doi: 10.1371/journal.pone.0021022 (PMC3120832; doi:10.1371/journal.pone.0021022)
Supplement: Table S2 — Characterization of gene fusion events. Gene symbol of the genes involved in each fusion event are given. The total number of reads reporting the fusion event is shown and also the number reads identified by each library is shown in parenthesis (C – C5.2 cells; H – Hb4a cells; U – reads of undefined origin). MH – Number of bases with microhomology between the genes. NT – Number of bases of short non-templated sequences. Genes involved in more than one event identified by our data are colored red. Genes involved in fusion events reported in the literature are shown, and the corresponding genes(s) reported are identified by their gene symbol. Light green highlights the events selected for validation. Bright green highlights validated gene fusions. (DOC) [file pone.0021022.s007.doc]

|  | **Genes** | **total number of reads (C:C5.2; H:Hb4a; U:undefined)** | **MH** | **NT** | **Human Genome Coordinates** | | | | | | **Gene Fusion reported in the literature** | | **Alignment at exon border** | |
| --- | --- | --- | --- | --- | --- | --- | --- | --- | --- | --- | --- | --- | --- | --- |
|  | **Gene 1: Gene 2** |  |  |  | **chr1** | **Start** | **End** | **chr2** | **Start** | **End** | **Gene 1** | **Gene 2** | **Gene 1** | **Gene 2** |
| Inter-chromossomic | TOMM20:PKM2 | 111 (88C;2H;21U) | 1 | 0 | chr1 | 233340301 | 233340373 | chr15 | 70278910 | 70279008 |  |  | NO | NO |
|  | F11R:KRT13 | 268 (233C;7H;28U) | 10 | 0 | chr1 | 159232770 | 159232851 | chr17 | 36912525 | 36912592 | GOLGB1 |  | NO | YES |
|  | ECHDC2:RAVER1 | 13 (5C;3H;5U) | 7 | 0 | chr1 | 53159132 | 53159218 | chr19 | 10288365 | 10288457 |  |  | NO | NO |
|  | RBM8A:SLC27A5 | 1 (1C) | 8 | 0 | chr1 | 144219039 | 144219431 | chr19 | 63701795 | 63702006 |  |  | YES | NO |
|  | LOC642587:RPL14 | 12 (6C;3H;3U) | 5 | 0 | chr1 | 207672278 | 207672346 | chr3 | 40478123 | 40478480 |  |  | NO | NO |
|  | GOT1:RAB8A | 29 (15C;11H;3U) | 2 | 0 | chr10 | 101146930 | 101147017 | chr19 | 16104517 | 16104582 |  |  | NO | NO |
|  | RPS3:FAM57A | 216 (203C;3H;10U) | 5 | 0 | chr11 | 74792709 | 74792834 | chr17 | 592055 | 592165 | ANK3 |  | YES | NO |
|  | RPS3:SMPD4 | 2 (1C;1H) | 8 | 0 | chr11 | 74793540 | 74794371 | chr2 | 130638426 | 130641526 | ANK3 |  | NO | NO |
|  | FTH1:COPB2 | 45 (36C;2H;7U) | 4 | 0 | chr11 | 61491399 | 61491513 | chr3 | 140574760 | 140574859 | ORAOV1 |  | NO | NO |
|  | RPLP2:PLEC1 | 33 (32C;1U) | 4 | 0 | chr11 | 800257 | 800312 | chr8 | 145072939 | 145072998 | AX747721, AX747721 |  | NO | YES |
|  | KRT5:TLN1 | 631 (425C;158H;48U) | 6 | 0 | chr12 | 51196827 | 51197200 | chr9 | 35687516 | 35687658 |  |  | NO | NO |
|  | KRT8:WDR45 | 923 (518C;291H;114U) | 0 | 2 | chr12 | 51579955 | 51580066 | chrX | 48820218 | 48820312 |  |  | NO | NO |
|  | TPT1:RPLP1 | 118 (103C;5U) | 5 | 0 | chr13 | 44813207 | 44813294 | chr15 | 67532330 | 67532414 |  | MST150 | NO | NO |
|  | KCTD5:NAP1L1 | 8 (6C;1H;1U) | 3 | 0 | chr16 | 2697445 | 2697512 | chr12 | 74749018 | 74764638 |  |  | NO | NO |
|  | SLC7A5:DNPEP | 2 (2C) | 6 | 0 | chr16 | 86422293 | 86422376 | chr2 | 219959305 | 219959368 |  |  | NO | NO |
|  | CLTC:C14orf147 | 4 (2C;2H) | 3 | 0 | chr17 | 55079717 | 55079809 | chr14 | 33974138 | 33974202 |  |  | YES | NO |
|  | ERBB2:RPS17 | 1401 (1241C;1H;159U) | 0 | 0 | chr17 | 35127201 | 35129613 | chr15 | 80611484 | 80611903 |  |  | YES | NO |
|  | BLMH:MKRN1 | 5 (2C;2H;1U) | 5 | 0 | chr17 | 25642453 | 25642580 | chr7 | 139799963 | 139800081 | CCDC117 |  | YES | NO |
|  | KRT13:SLC25A37 | 1054 (836C;156H;62U) | 6 | 0 | chr17 | 36913112 | 36913206 | chr8 | 23479589 | 23479665 |  |  | NO | NO |
|  | TAF15:HUWE1 | 4 (1C;2H;1U) | 7 | 0 | chr17 | 31173872 | 31173948 | chrX | 53582111 | 53582547 |  | PIG-T, ATP5A1, ATP5A1, HCK | YES | NO |
|  | **SCAF1:VAMP8** | **29 (26C;3U)** | **9** | **0** | **chr19** | **54852861** | **54852958** | **chr2** | **85658182** | **85659666** |  |  | NO | NO |
|  | CNOT3:TMED9 | 27 (18C;8H;1U) | 6 | 0 | chr19 | 59341509 | 59342205 | chr5 | 176955359 | 176955481 |  |  | YES | NO |
|  | C3:ACTB | 565 (491C;12H;62U) | 6 | 0 | chr19 | 6637209 | 6637767 | chr7 | 5534193 | 5534274 | GALNT6 |  | NO | NO |
|  | NLRP2:AKR1B1 | 23 (15C;4H;4U) | 14 | 0 | chr19 | 60189469 | 60193236 | chr7 | 133777866 | 133777932 |  |  | NO | NO |
|  | PTPN18:ENTPD6 | 2 (1H;1U) | 6 | 0 | chr2 | 130847502 | 130847612 | chr20 | 25155213 | 25155348 |  |  | NO | NO |
|  | ATP5G3:SDCBP | 287 (249C;3H;35U) | 10 | 0 | chr2 | 175754328 | 175754408 | chr8 | 59653222 | 59653306 |  |  | YES | NO |
|  | DIAPH1:SHMT1 | 36 (25C;7H;4U) | 0 | 0 | chr5 | 140875692 | 140875795 | chr17 | 18174686 | 18177304 |  |  | NO | NO |
|  | CLTB:RPL18 | 1336 (1241C;8H;87U) | 0 | 0 | chr5 | 175757221 | 175757628 | chr19 | 53812384 | 53814233 |  | BC051359 | NO | NO |
|  | CAMLG:ZFP36L2 | 3 (3C) | 7 | 0 | chr5 | 134115563 | 134115641 | chr2 | 43306377 | 43306968 |  |  | NO | NO |
|  | STK17A:RPS15 | 194 (98C;87H;9U) | 0 | 0 | chr7 | 43602220 | 43614501 | chr19 | 1391355 | 1391489 |  |  | YES | NO |
|  | TXN:AFTPH | 8 (6C;1H;1U) | 3 | 0 | chr9 | 112046878 | 112052948 | chr2 | 64632098 | 64632206 |  | SP3 | YES | NO |
|  | **EIF5A:FTH1** | **68 (53C;2H;13U)** | **2** | **0** | **chr17** | **7151656** | **7151698** | **chr11** | **61491398** | **61491503** |  | **ORAOV1** | **NO** | **NO** |
|  | ATF4:STRAP | 112 (42C;50H;20U) | 0 | 0 | chr22 | 38248486 | 38248634 | chr12 | 15939596 | 15939672 |  |  | YES | NO |
| Intra-chromossomic | FAM36A:CAP1 | 42 (27C;9H;6U) | 1 | 0 | chr1 | 243072967 | 243073066 | chr1 | 40310379 | 40310472 |  |  | YES | NO |
|  | **CDH13:MLYCD** | **1 (1C)** | **0** | **0** | **chr16** | **82269310** | **82269355** | **chr16** | **82503415** | **82503473** | **MIAT** |  | **YES** | **YES** |
|  | RSL24D1:RAB27A | 5 (4C;1U) | 4 | 0 | chr15 | 53276297 | 53276395 | chr15 | 53308140 | 53309978 |  |  | YES | YES |
|  | STAT6:TPI1 | 2 (1C;1U) | 13 | 0 | chr12 | 55775569 | 55775628 | chr12 | 6848683 | 6848772 |  | TOP2B | NO | NO |
|  | RPLP1:NIPA2 | 999 (935C;64U) | 0 | 0 | chr15 | 67532272 | 67532414 | chr15 | 20556961 | 20557068 | MST150 |  | NO | NO |
|  | GEMIN4:CANT1 | 3 (1C; 2H) | 7 | 0 | chr17 | 597602 | 597724 | chr17 | 74501361 | 74501520 |  |  | NO | YES |
