# Supplementary material for: Poly (A)+ Transcriptome Assessment of ERBB2-Induced Alterations in Breast Cell Lines
Source: PLoS One. 2011 Jun 22;6(6):e21022. doi: 10.1371/journal.pone.0021022 (PMC3120832; doi:10.1371/journal.pone.0021022)
Supplement: Table S3 — Alternative splicing events detected for each cell line. The number of alternative splicing events detected for each sample normalized by the total number of reads generated for each cell line. (DOC) [file pone.0021022.s008.doc]

| **AS event type** | **HB4a** | **C5.2** |
| --- | --- | --- |
| intron retention | 509.76 | 492 |
| exon inclusion | 185.26 | 243 |
| exon skipping | 152.22 | 249 |
| alternative aceptor donor | 146.32 | 249 |
