# Supplementary material for: Poly (A)+ Transcriptome Assessment of ERBB2-Induced Alterations in Breast Cell Lines
Source: PLoS One. 2011 Jun 22;6(6):e21022. doi: 10.1371/journal.pone.0021022 (PMC3120832; doi:10.1371/journal.pone.0021022)
Supplement: Table S4 — Alternative splicing variants modulated by ERBB2 expression. The position of the novel exon identified is shown according to the number of the flanking exons. The expression level obtained by qRT-PCR is reported as fold-change between C5.2 and HB4a. (DOC) [file pone.0021022.s009.doc]

| Gene Symbol | Position | Inclusion between exons | Size of novel exon | Reported by EST | Fold-change  C5.2/HB4a |
| --- | --- | --- | --- | --- | --- |
| *CLTC* | CDS | 25 and 26 | 21bp | YES | 3 |
| *CSRP2BP* | CDS | 8 and 9 | 47bp | NO | 2 |
| *KIAA1033* | CDS | 26 and 27 | 115bp | NO | 4 |
| *NR2C1* | CDS | 2 and 3 | 38bp | YES | 2 |
| *RPS19* | CDS | 3 and 4 | 31bp | NO | 3 |
| *PRCC* | CDS | 5 and 6 | 134bp | NO | 3 |
