# Supplementary material for: Poly (A)+ Transcriptome Assessment of ERBB2-Induced Alterations in Breast Cell Lines
Source: PLoS One. 2011 Jun 22;6(6):e21022. doi: 10.1371/journal.pone.0021022 (PMC3120832; doi:10.1371/journal.pone.0021022)
Supplement: Table S5 — Validation of differential gene expression modulated by ERBB2 . The mRNA seq data is given as a fold-change between C5.2 and HB4a cell lines. When no reads were identified in the RNA-seq from one of the cell lines, we calculated fold-change by replacing “0” by “1”. Positive and negative values correspond to higher expression in C5.2 and HB4a, respectively. The qRT_PCR results are given as fold-change obtained by 2−ΔΔCT. In grey: genes validated in the qRT-PCR by the criterion for differentially expressed genes as Fold-change>2. (DOC) [file pone.0021022.s010.doc]

|  | **RNA seq** | | | **qRT_PCR** |
| --- | --- | --- | --- | --- |
| **Gene Symbol** | **C5.2** | **Hb4a** | **Fold change** | **Fold change** |
| *ALDH2* | 14 | 0 | 14 | 41 |
| *ALDOA* | 1919 | 458 | 4 | 1 |
| *ANGPTL4* | 246 | 7 | 35 | 7 |
| *ANXA6* | 13 | 0 | 13 | 2 |
| *ATP5G3* | 685 | 14 | 49 | 2 |
| *ATP5L* | 568 | 55 | 10 | 2 |
| *C12orf44* | 146 | 14 | 10 | 2 |
| *CAPG* | 720 | 96 | 8 | 1 |
| *CDC20* | 392 | 89 | 4 | 1 |
| *COL3A1* | 28 | 0 | 28 | 493 |
| *COPE* | 170 | 27 | 6 | 1 |
| *COX11* | 20 | 0 | 20 | 2 |
| *COX4I1* | 562 | 14 | 40 | 2 |
| *COX8A* | 1293 | 294 | 4 | 1 |
| *CSDA* | 199 | 14 | 14 | 2 |
| *DST* | 35 | 212 | -6 | 1 |
| *EEF1A1* | 4781 | 96 | 50 | -1 |
| *EEF1B2* | 263 | 48 | 5 | 1 |
| *EIF4EBP1* | 199 | 41 | 5 | 1 |
| *ERBB2IP* | 18 | 137 | -8 | 1 |
| *FAU* | 158 | 14 | 11 | 1 |
| *FBXL6* | 26 | 0 | 26 | 2 |
| *GALNT3* | 6 | 267 | -45 | -2 |
| *HMGA1* | 281 | 27 | 10 | 2 |
| *HMGB1* | 556 | 82 | 7 | 2 |
| *HMGB2* | 20 | 0 | 20 | 1 |
| *HMGN2* | 374 | 7 | 53 | 1 |
| *HRAS* | 257 | 41 | 6 | 2 |
| *HSPA8* | 544 | 75 | 7 | 4 |
| *HSPE1* | 445 | 7 | 64 | 2 |
| *IL6ST* | 6 | 226 | -38 | 1 |
| *JUP* | 23 | 68 | -3 | 1 |
| *KRT15* | 1129 | 164 | 7 | 3 |
| *KRT19* | 9515 | 2345 | 4 | 2 |
| *KRT4* | 14 | 0 | 14 | 52 |
| *KRT6A* | 492 | 96 | 5 | 3 |
| *KRT7* | 11141 | 2297 | 5 | 2 |
| *LAMB1* | 105 | 540 | -5 | 1 |
| *LAMC1* | 222 | 971 | -4 | 2 |
| *LAMC2* | 146 | 608 | -4 | 1 |
| *LMNA* | 339 | 48 | 7 | 2 |
| *LOX* | 12 | 171 | -14 | -8 |
| *LRPAP1* | 35 | 0 | 35 | 1 |
| *MME* | 0 | 27 | -27 | -11 |
| *NDUFA1* | 427 | 14 | 31 | 1 |
| *NDUFA13* | 948 | 157 | 6 | 1 |
| *NDUFA2* | 275 | 21 | 13 | 1 |
| *NDUFA5* | 13 | 0 | 13 | 1 |
| *NDUFB3* | 23 | 0 | 23 | 2 |
| *NDUFB8* | 1001 | 123 | 8 | 2 |
| *NDUFS7* | 334 | 14 | 24 | 1 |
| *NDUFS8* | 211 | 41 | 5 | 1 |
| *PDS5B* | 0 | 26 | -26 | 1 |
| *PFKP* | 749 | 123 | 6 | 4 |
| *PHB* | 492 | 75 | 7 | 3 |
| *PHB2* | 4166 | 827 | 5 | 3 |
| *PTMA* | 46 | 0 | 46 | 1 |
| *PTMS* | 427 | 14 | 31 | 1 |
| *RAN* | 351 | 21 | 17 | 2 |
| *RPL10A* | 2949 | 595 | 5 | 1 |
| *RPL29* | 1229 | 533 | 2 | 2 |
| *RPL31* | 152 | 14 | 11 | 2 |
| *RPL38* | 1006 | 96 | 10 | 1 |
| *RPL39* | 1375 | 321 | 4 | 2 |
| *RPL41* | 1042 | 21 | 50 | 2 |
| *RPL8* | 1691 | 48 | 35 | 3 |
| *RPLP1* | 2475 | 41 | 60 | 2 |
| *RPS13* | 860 | 103 | 8 | 1 |
| *RPS15A* | 714 | 68 | 11 | 1 |
| *RPS19* | 6267 | 1306 | 5 | 1 |
| *RPS24* | 363 | 62 | 6 | 2 |
| *RPS27A* | 35 | 0 | 35 | 1 |
| *RPS6* | 170 | 7 | 24 | 1 |
| *SEMA3C* | 25 | 0 | 25 | 1 |
| *SFN* | 374 | 7 | 53 | 1 |
| *SLC3A2* | 257 | 21 | 12 | 3 |
| *SOS2* | 6 | 301 | -50 | 1 |
| *SOX15* | 15 | 0 | 15 | 1 |
| *STAT3* | 64 | 390 | -6 | 1 |
| *TGFBR3* | 6 | 246 | -41 | 1 |
| *TIMP1* | 357 | 7 | 51 | 2 |
| *TPI1* | 930 | 34 | 27 | 2 |
| *TUBB2C* | 568 | 41 | 14 | 2 |
| *TXNIP* | 275 | 2639 | -10 | -3 |
| *TXNRD2* | 146 | 14 | 10 | 2 |
| *UCRC* | 30 | 0 | 30 | 2 |
| *UQCRB* | 556 | 34 | 16 | 2 |
| *VEGFA* | 23 | 185 | -8 | 1 |
