# Supplementary material for: Poly (A)+ Transcriptome Assessment of ERBB2-Induced Alterations in Breast Cell Lines
Source: PLoS One. 2011 Jun 22;6(6):e21022. doi: 10.1371/journal.pone.0021022 (PMC3120832; doi:10.1371/journal.pone.0021022)
Supplement: Table S6 — Effects of rapamycin treatment on genes influenced by ERBB2 -mediated expression. The results from quantitative RT-PCR on rapamycin-treated cell lines for the 46 validated genes are given as fold-change between C5.2 and HB4a cell lines. Positive and negative values correspond to higher expression in C5.2 and HB4a cell lines, respectively. The fourth columns show the qRT_PCR results obtained from the C5.2 and HB4a cell lines and from the cells lines after rapamycin treatment. Response to rapamycin was considered when a decrease or inversion of fold-change between C5.2 and HB4a cell lines compared to non-treated cell lines was observed. Yes and No represent response or no response to rapamycin, respectively. (nd) Cycle threshold not determined. (DOC) [file pone.0021022.s011.doc]

| **Gene Symbol** | **mRNA seq** | **Same cell lines** | **After Rapamycin treatment** | |
| --- | --- | --- | --- | --- |
| *ALDH2* | 14 | 41 | nd | |
| *ANGPTL4* | 35 | 7 | 5 | NO |
| *ANXA6* | 13 | 2 | 2 | NO |
| *ATP5G3* | 49 | 2 | -2 | YES |
| *ATP5L* | 10 | 2 | 6 | NO |
| *C12orf44* | 10 | 2 | 1 | YES |
| *COL3A1* | 28 | 493 | nd | |
| *COX11* | 20 | 2 | nd | |
| *COX4I1* | 40 | 2 | -2 | YES |
| *CSDA* | 14 | 2 | 3 | NO |
| *FBXL6* | 26 | 2 | 1 | YES |
| *GALNT3* | -45 | -2 | nd | |
| *HMGA1* | 10 | 2 | 1 | YES |
| *HMGB1* | 7 | 2 | 3 | NO |
| *HRAS* | 6 | 2 | -2 | YES |
| *HSPA8* | 7 | 4 | 3 | NO |
| *HSPE1* | 64 | 2 | 2 | NO |
| *KRT15* | 7 | 3 | -3 | YES |
| *KRT19* | 4 | 2 | nd | |
| *KRT4* | 14 | 52 | nd | |
| *KRT6A* | 5 | 3 | -1620 | YES |
| *KRT7* | 5 | 2 | -7 | YES |
| *LMNA* | 7 | 2 | 1 | YES |
| *LOX* | -14 | -8 | -2 | YES |
| *MME* | -27 | -11 | nd | |
| *NDUFB3* | 23 | 2 | 1 | YES |
| *NDUFB8* | 8 | 2 | -2 | YES |
| *PFKP* | 6 | 4 | 1 | YES |
| *PHB* | 7 | 3 | 1 | YES |
| *PHB2* | 5 | 3 | -3 | YES |
| *RAN* | 17 | 2 | -1 | YES |
| *RPL29* | 2 | 2 | -2 | YES |
| *RPL31* | 11 | 2 | 1 | YES |
| *RPL39* | 4 | 2 | 1 | YES |
