# Supplementary material for: Poly (A)+ Transcriptome Assessment of ERBB2-Induced Alterations in Breast Cell Lines
Source: PLoS One. 2011 Jun 22;6(6):e21022. doi: 10.1371/journal.pone.0021022 (PMC3120832; doi:10.1371/journal.pone.0021022)
Supplement: Table S7 — Validation of gene Fusion. The gene fusions evaluated are characterized by the 2 chromosomes involved in the event as well as the corresponding genes. The specific hybridization sequence of the probes and the amplicon size expected after PCR are shown. (DOC) [file pone.0021022.s012.doc]

| **Chromosome** | **Gene Symbol** | **Probe Hibridization Sequence** | **PCR primers** |
| --- | --- | --- | --- |
| Chromosome 22 | ATF4 | GAGGATAGTCAGGAGC | GTGAGTGCAAAGAGCTGG |
| Chromosome 12 | STRAP | ATCGTCCATAAGTTATAACC | GATCGTCCATAAGTTATAACC |
| Chromosome X | KRT8 | CATAGCCTGTGTGTCTC | CTCGTCTGCTCCATTCAC |
| Chromosome 12 | WDR45 | CTCTCAGCCTCAGCC | CTCTGCAGCTCCTCATAC |
| Chromosome 11 | RPLP2 | CTTCTTGATGTCCTTGGC | GTTGAGCCGGTCGTCG |
| Chromosome 8 | PLEC1 | CCAGTACATCAAGTTCATC | CGTGGACCTGCGTAC |
| Chromosome 11 | FTH1 | GTTGATGGCGGCCTC | GGACAGGTAAACGTAGGAG |
| Chromosome 17 | EIF5A | GCGCCTGCGTACTAAG | GAGGAGATAGATAGCACGC |
| Chromosome 8 | SDCBP | CTGTGCAGGATGGAGC | CCAAGTACTTCAGATCAATGG |
| Chromosome 2 | ATP5G3 | GTGCAGGCGAGCTTG | CTCTGGTCGAGATAACACTG |
| Chromosome 19 | SLC27A5 | GGGTAGCGTAGGCAGG | GTCCAGTACAAACAGAGGG |
| Chromosome 1 | RBM8A | GAGCCAAAGCCGCGACC | GCGAGATGGCGGACG |
| Chromosome 14 | C14orf147 | CAATATCGCCATGATGTGCTG | CATTCCAACTTCGTAGGGTG |
| Chromosome 17 | CLTC | TGCAGATATACCAGTCAGAAGTAAC | GAGTATGGAAGGAGAGTCTC |
| Chromosome 7 | STK17A | CAGCTAATAAAGGCGCACATGAC | GAGATGATCGGCCACTAC |
| Chromosome 19 | RPS15 | CACGAGTGTGTAAAAAGTGAACAC | CATGAGATTGCTGTACTTGAAC |
| Chromosome 17 | SHMT1 | GATAAACTTTAAATTCCAGAGTCATAGC | CCCTTCCACCATCTGTG |
| Chromosome 5 | DIAPH1 | CTGGACTATGGGTTGTACTGTG | GAACTCTGTAACCTGCCAC |
| Chromosome 2 | VAMP8 | CCAGCTTCACCTCCTCCACC | GTGGCAGATCTTGTGGAC |
| Chromosome 19 | SACF1 | TTCACTGGCTTCCTCCATGTCTC | GCAGGCTGAAAGTTGGAG |
| Chromosome 9 | TXN | CAACATTCCAGTTTTTAAGAAGGGACA | CATTCCCTCTCTGAAAAGTATTC |
| Chromosome 2 | AFTPH | AAAGCAACTGAACAATGGAGCCAG | CAACAAACCCTACACCAGAAG |
| Chromosome 1 | FAM36A | GCTTTGCATAATTATACCTACAATGAA | GTGGTTGATCTTAACTTCG |
| Chromosome 1 | CAP1 | GTGAGATAGGAGAGTCTCTGG | CTCTATAGGAAGGTGCTG |
| Chromosome 17 | CANT1 | GCCATGCAGAACTAGAGCGCT | GACACAGAGACACGGTG |
| Chromosome 17 | GEMIN4 | ACAGCGAGAAGGACGACGAG | CACAATGATCTGGTCGTC |
| Chromosome 16 | MLYCD | CAGAAATTTCATAGTCCAATGGCTG | GATGTGGACGGTGGC |
| Chromosome 16 | CDH13 | CAACTCCTTGACGACTCGC | CATCAGAAACAGAAGAGAAG |
