# Supplementary material for: Poly (A)+ Transcriptome Assessment of ERBB2-Induced Alterations in Breast Cell Lines
Source: PLoS One. 2011 Jun 22;6(6):e21022. doi: 10.1371/journal.pone.0021022 (PMC3120832; doi:10.1371/journal.pone.0021022)
Supplement: Table S8 — Novel alternative splicing variants. The forward and reverse primer sequences used for each validation are shown with the corresponding gene symbol and the amplicon size. (DOC) [file pone.0021022.s013.doc]

| **Gene** | **Foward Primer** | **Reverse Primer** | **Amplicon** |
| --- | --- | --- | --- |
| *AET1G* | CAGGTGTGTGCGAACAG | CTTCAGCTCAATCCCAATC | 89 |
| *APEX1* | GTTTGTCATTCCCTTGATG | CTCCTGCTGCCTCTTTGTC | 83 |
| *BC039445* | GTCGACCTCGCAACAG | CCTATGAAATAGTCTCGGC | 116 |
| *CLTC* | CCTAGAAACTGCATGGAG | CTTTTCCTTTATTGCATCAAC | 103 |
| *CSRP2BP* | GATTCATCCTGTTTTGCTTCTG | CCTTTGGCTTCATGGTTCC | 131 |
| *CUEDC2* | GGATTACAGGCATGAACC | GATGAGAGCTGCACCG | 101 |
| *FLJ00150* | CTCAGAAAGGGATAGTAGC | GTAGCCCAGGACAACCATG | 104 |
| *FTJ3* | GTCTGCTGCACTCATATCC | CCTGGCATCAAGCAATCC | 95 |
| *KIAA1033* | CATTCGTACATAGCCCATAGC | CAGTAAACAAAGTCTCTGTCC | 92 |
| *KIF2A* | GCAACAGCAAGAACTTAGAG | CTTCTAGGAAATAATACCACC | 74 |
| *NR_002599* | GCGAAGAGCCGTTAGTC | GAAAACAGAATTCAAGCTACTG | 115 |
| *NR2C1* | CAAGTGCTGTCACAATCTG | GTGGCAATAGAATCGGTAC | 79 |
| *PAWR* | CCACCTAGAACAGTTTCAG | CATTCTCTTCACCCTCCAAC | 104 |
| *PDE6D* | CCATGTGCCAAGTGAGTG | CCTAACTCCACAAATACCTG | 103 |
| *PP2R2A* | CTTTCAAGTTATACCCTTCTGG | GTATAGTGGAGAAGCCTGG | 129 |
| *PRCC* | CACCTAGTAGCTGAGAACAG | GTTGGCTGCTCACCTTTC | 123 |
| *RPLP1* | CATGGCCTCTGTCTCC | GGCAATTACACCGAAAGAG | 108 |
| *RPS19* | GTTCATCTTTCAGTCCTCAG | GCTTGCTCCCTACGATG | 106 |
| *RWDD1* | GAAAGCCAAGTTTGATG | CTCTTTTTCTGTGAATTTC | 114 |
| *ZNF567* | GCTCAGAAGACTCTATATATGG | CTGGGTAAGTGAAGACAC | 124 |
