# Supplementary material for: Poly (A)+ Transcriptome Assessment of ERBB2-Induced Alterations in Breast Cell Lines
Source: PLoS One. 2011 Jun 22;6(6):e21022. doi: 10.1371/journal.pone.0021022 (PMC3120832; doi:10.1371/journal.pone.0021022)
Supplement: Table S9 — Putative novel genes. The chromosome localization of each putative novel gene is shown as well as the sequences of forward and reverse primers and the respective amplicon size. (DOC) [file pone.0021022.s014.doc]

| **Chrom. location** | **Foward Primer** | **Reverse Primer** | **Amplicon** |
| --- | --- | --- | --- |
| 5p13.3 | CCCACTTTGGTCTCCC | CTGCTTACAGTTCTTCATGC | 147 |
| 6q23.2 | TATATCGAATATTGTTAATAG | TTCACTGCAGTCTGG | 182 |
| 20q13.13 | CACGCCACTGCACTCC | CCTGACCTTTGTACATGCTG | 144 |
| 9q21.13 | CCTTCCATCTCAGCCTCC | CAGGAAGCTGGTATTCAAGAG | 170 |
| 20p12.1 | GATCAAAGAAGCCTCTGC | CACACCATACATGCTCTTC | 240 |
| 1q32.2 | GGTTCTAGTTTTGGTTCTTC | GGCTTATCTCTGTTGAATC | 147 |
